# Mitigating surface noise and attenuation in continuous monitoring of geologically stored CO_2_ using a borehole portable active seismic source

**DOI:** 10.1038/s41598-025-19824-8

**Published:** 2025-10-14

**Authors:** Ahmad B. Ahmad, Takeshi Tsuji, Kazuyuki Tani, Yuta Mizutani, Toru Sano

**Affiliations:** 1 School of Engineering, The University of Tokyo, 7-3-1 Hongo, Bunkyo-Ku, Tokyo, Japan; 2 International Institute for Carbon-Neutral Energy Research, Kyushu University, Nishi-ku, Fukuoka, Japan; 3 ENEOS Xplora Inc., 1-3-1 Azabudai, Minato-ku, Tokyo, Japan

**Keywords:** Borehole active seismic source, Carbon capture and storage monitoring, Optimal monitoring schedule, Continuous monitoring, Environmental noise mitigation, Geophysics, Seismology, Carbon capture and storage

## Abstract

We investigate strategies to mitigate the impact of environmental noise and surface influences in long-term monitoring of subsurface reservoirs using the borehole-deployable Portable Active Seismic Source (B-PASS). Unlike traditional surface seismic sources, the B-PASS system minimizes the impact of surface environmental factors such as temperature fluctuations and groundwater variations. This capability makes it particularly effective for long-term applications, especially CO_2_ storage monitoring. Field tests of B-PASS were conducted in a gas field underlain by a shallow, highly attenuating beach sand. This study tested two source configurations: vertical motion and horizontal motion. By stacking the repeatable signals from B-PASS, we achieved effective noise reduction and enhanced the quality of the seismic signal. The source remained deployed in the borehole for 10 days; stability was quantitatively assessed over 7 days comprising approximately 8,000 sweeps., with stacking providing an hourly temporal resolution. The results demonstrated significant signal propagation to the farthest receiver, located approximately 450 m away, even through a highly attenuating layer. Deeper source deployment proved advantageous, enhancing the signal-to-noise (S/N) ratio for P-waves by up to five times compared to a surface source. The field experiments further demonstrated that environmental noise significantly influenced signal propagation, and midnight was identified as the optimal operational period due to less ambient noise. Therefore, by considering the daily temporal variation of the S/N ratio of the field before the long-term monitoring, we can identify the optimal operation time of the monitoring. To determine the optimal number of signal stacks, we developed a method to evaluate how the S/N ratio varies with stacking number and offset (source–receiver distance). Based on these results, the B-PASS demonstrates its potential as a reliable solution for continuous monitoring, even in geologically challenging environments with surface attenuation and noise, by optimizing the daily monitoring schedule and the number of signal stacks.

**Supplementary Information:**

The online version contains supplementary material available at 10.1038/s41598-025-19824-8.

## Introduction

The reduction of CO_2_ emissions through carbon capture and storage (CCS) is increasingly recognized as a viable strategy for mitigating climate change^1,2^. Secure storage of CO_2_ in geological formations is essential, but accurate monitoring of injected CO_2_ with high spatiotempral resolution remains a significant challenge. A monitoring system must detect potential CO_2_ leaks, optimize storage operations by tracking CO_2_ migration and pressure changes, and minimize risks such as injection-induced seismicity^3^. Moreover, reliable monitoring can enhance public confidence in CCS by ensuring consistent oversight of storage fields^4^. A widely recognized geophysical method for CCS monitoring is the time-lapse or 4D seismic survey, which tracks spatiotemporal changes in seismic velocity to identify variations in CO_2_ saturation^5,6^. These techniques have been successfully applied in large-scale CCS projects like Weyburn-Midale, Quest, In Salah, and Sleipner, as well as demonstration or pilot projects such as Tomakomai, Ketzin, and Otway^7–9^. Recent advances in surface-based seismic methods, such as dual-element landstreamer and wireless systems, have improved CO_2_ site characterization in onshore settings^10^. While the conventional 4D seismic surveys used in the CCS projects have proven effective, they are typically conducted infrequently due to their high operational costs, which limits the temporal resolution for detecting sudden changes^11–14^, such as rapid CO_2_ leakage.

**Fig. 1:**
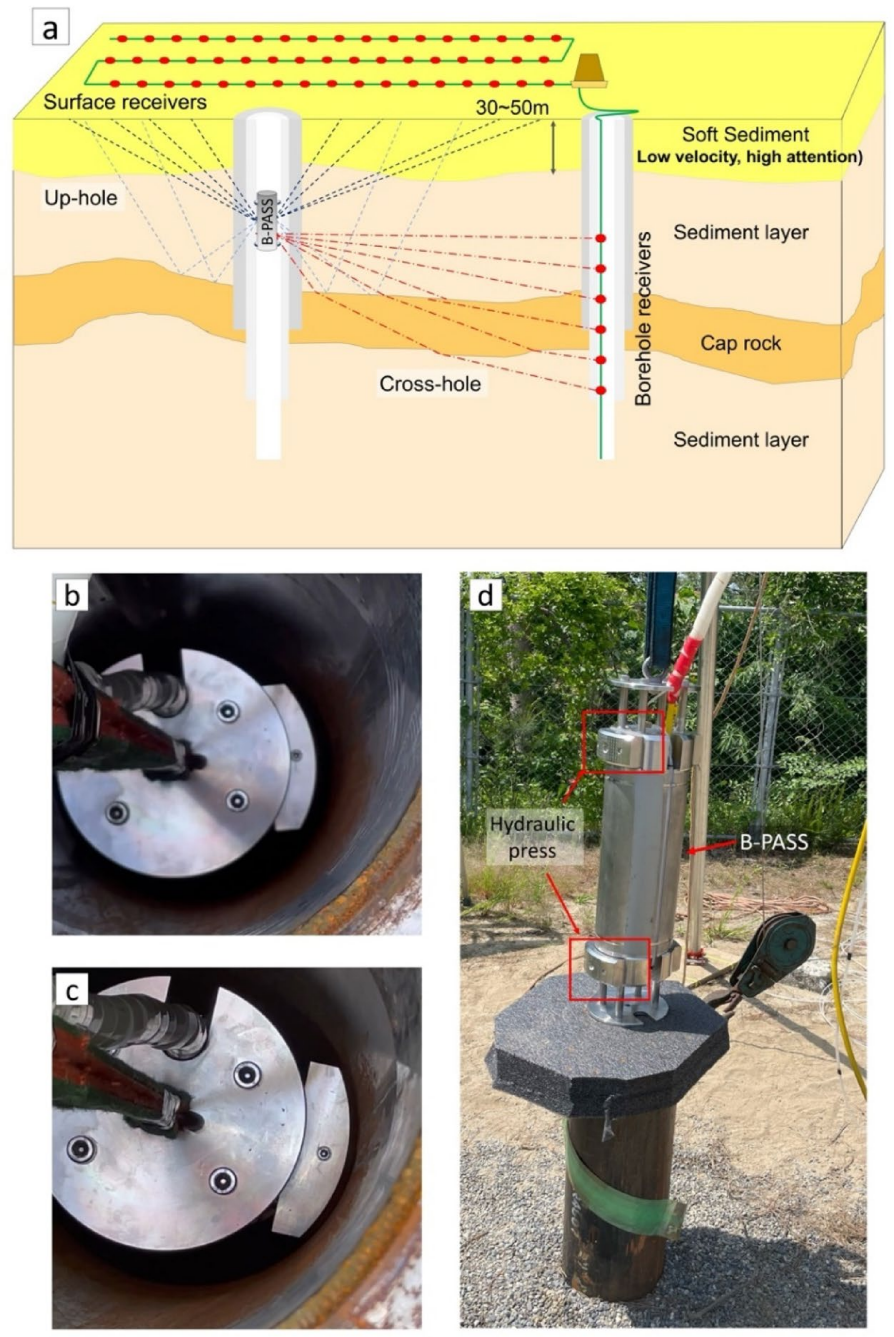
Schematic of B-PASS monitoring configurations. (**a**) Up-hole and cross-hole surveys with illustrations of the conceptual geological layers. Pictures of hydraulic coupling mechanism (**b**) before locking and (**c**) after locking inside the borehole. (**d**) Picture of B-PASS ready for deployment. The Schematic image in (**a**) was created using Microsoft PowerPoint software in Microsoft 365 (Version 16.0.1; https://www.microsoft.com/en-us/microsoft-365/powerpoint).

To achieve high-temporal-resolution monitoring of injected CO_2_, a continuous monitoring approach has been developed using an alternative, permanent-type seismic source^15,16^. Recent studies have demonstrated that even a small seismic source, termed the ‘Portable Active Seismic Source’ (PASS), can propagate monitoring signals over kilometer-scale distances^17^. The PASS system could address the need for a continuous and cost-effective monitoring solution. Unlike traditional impulsive sources, PASS offers a permanent or semi-permanent installation that allows for on-demand or continuous data acquisition, filling the temporal gap left by conventional surveys and enabling the tracking of dynamic reservoir processes in near real-time, and provides immediate information on reservoir properties at a fraction of the operational cost. The PASS system was inspired by the design of the accurately controlled, routinely operated signal system (ACROSS), mainly used for large-scale earthquake faults and volcanoes^16,18–21^. While maintaining the core functions of ACROSS, PASS is more compact and cost-effective. The system enhances the signal-to-noise ratio (S/N ratio) through the stacking of repeated signals. If we stacked the highly repeatable source waveforms, the signal from the PASS with a 10 kg eccentric mass can propagate close to 100 km^16^, and the PASS with a 10 g eccentric mass can propagate close to 1 km^17^. Still, PASS faces challenges when deployed at the surface, where environmental factors such as rain, groundwater fluctuations, and temperature extremes can affect its performance^17^. To overcome these challenges, we introduce a subsurface-deployed PASS, referred to as the Borehole-PASS (B-PASS). Deploying the PASS system underground offers significant advantages, such as immunity to weathered layers, water-level fluctuations, and surface noise, which can compromise source stability and monitoring accuracy. Moreover, the B-PASS has potential applications in offshore environments, where conventional PASS systems are impractical due to the presence of soft seafloor sediments (Fig. 1a).

Borehole seismic monitoring has been used for its potential to mitigate wave attenuation in soft, shallow geological formations. By operating closer to the target formation (i.e., injected CO_2_), they can improve the clarity of reflection signals and enhance spatial resolution, offering some advantages over surface-based seismic sources^22–24^. In addition, the B-PASS can reduce the influence of surface waves on the monitoring signals (i.e., body waves), as source excitation at greater depths does not generate significant surface waves. Techniques such as up-hole and cross-hole seismic surveys have demonstrated the effectiveness of borehole sources for subsurface investigations. For example, a study conducted at the Liso Field in Nigeria used an up-hole seismic survey, where dynamite explosions within boreholes generated seismic signals^25^. In addition, cross-hole seismic surveying, which involves measuring seismic waves between boreholes, provides high-definition data with greater resistance to surface noise. By generating seismic waves in one borehole and detecting them in adjacent boreholes, this method is highly effective for detailed subsurface investigations, particularly in complex geological settings^26^. However, traditional borehole sources, including mechanical, electromechanical, and explosive devices, often risk damaging borehole casings and compromising well integrity if used repeatedly^27–29^; this issue prevents the use of boreholes for long-term monitoring applications. Most recent borehole monitoring efforts have focused on passive sensing techniques, such as microseismic noise analysis^30^ or non-seismic methods, like cross-borehole resistivity imaging^31^. Despite the growing importance of continuous monitoring in CO₂ storage operations, a lack of practical, deployable seismic geophysical methods remains for long-term monitoring within boreholes.

The B-PASS addresses these limitations by offering a compact, low-impact solution with long-term operational stability and high reliability. Field tests conducted in a highly attenuating layer at Nakajo, Niigata, Japan, demonstrate the system’s effective performance in terms of signal propagation, stability, S/N ratio with depth, and resistance to environmental noise. Additionally, strategies for optimizing PASS operation are explored, including identifying optimal conditions for minimizing noise interference, sustainable deployment configurations, and enhancing temporal resolution for continuous monitoring. These technological advancements indicate that the B-PASS system has strong potential as a practical tool for continuous CCS monitoring, and may further contribute to a wide range of geophysical investigations^32^.

## Borehole-PASS

The cylindrical shape of the PASS for borehole deployment was first introduced in 2023^33^. In this study, we utilized the cylindrical shape PASS system with an eccentric mass of 0.16 kg mounted on a disk of 4 cm radius, generating a force of 632 N during 50 Hz rotation. The rotational force of the motor is transmitted to the eccentric weights via bevel gears. The B-PASS system generates a linear chirp signal, with the frequency sweeping continuously across the operational range (e.g., 5–70 Hz) over 30 s. By stacking these chirp signals, the S/N ratio of the seismic data is enhanced, thus improving the quality of the transfer function. Finally, the transfer function is computed by cross-correlating the stacked signals recorded at different seismic stations with the source function of the B-PASS system.

The orientation of the rotation axis of the eccentric mass in the source system plays a critical role in controlling the direction of the generated central force^34^. Two B-PASS designs were developed to optimize the force in specific directions. The first design, optimized for vertical motion, employs two axes connected by a gear mechanism^33^, which suppresses unwanted horizontal movement while enhancing vertical motion along the borehole axis. As a result, it primarily generates P-waves and SV-waves. In contrast, the second design, optimized for horizontal motion, uses multiple masses that rotate around the vertical axis in the same direction. Thus, incorporating the positional information of the eccentric mass into the analytical framework allows the excitation of the desired orientation of the horizontal force. These unique configurations allow the B-PASS system to be adapted to various monitoring needs and requirements.

The B-PASS system is coupled with the borehole casing using a hydraulic mechanism that applies water pressure to securely press it against the borehole’s inner wall (Fig. 1b-c). This study marks the first successful operation of the PASS in the borehole, enabled by the hydraulic coupling. We also achieved the longest operational period to date, with the B-PASS system positioned in the borehole for a continuous 10-day period. This study does not focus on the new PASS design itself but rather explores how this design is suitable for monitoring applications when buried at various depths, and enhancing the S/N ratio of body waves while reducing surface interference, which is crucial for effective monitoring.

The key advantage of this mechanical design, which uses a motor to rotate an eccentric mass, is the ability to generate low-frequency signals by starting its motion from a static position. As demonstrated in our field experiments (Table 1), this configuration enables stable operation at frequencies as low as 5 Hz. The design, focusing on a non-impulsive, low-frequency (5–70 Hz) continuous vibration, was chosen to ensure long-term borehole integrity, thereby overcoming a common limitation of traditional sources. It stands in contrast to other sources used in CCS monitoring, such as the SPH-54 piezoelectric source, which operates as a high-frequency (350–3500 Hz) swept-impact device^35,36^. The high-repetition, impact-based nature of such sources has been linked to cumulative fatigue damage and deteriorating near-borehole conditions in a long-term monitoring project^35^. The B-PASS’s non-impulsive, low-frequency approach is therefore specifically designed to be a safe and stable tool for continuous seismic monitoring. Other specialized systems, such as the Sandia hydraulic source^29^, have demonstrated the viability of deep borehole operations. In this study, we validate whether the motor-driven source technology (i.e., PASS), already proven for surface monitoring, can be adapted to deliver high-repeatability performance in a borehole and mitigate the surface noise.

**Table 1:** Operation and parameter settings of the horizontal-motion B-PASS system.

Source depth	Sweeps	Frequency	Start time (JST)	End time (JST)
0.5 m	104	10–60 Hz	2022-11-17 4:35 pm	2022-11-17 6:21 pm
	518	10–50 Hz	2022-11-17 18:24 pm	2022-11-18 3:01 pm
	30	5–60 Hz	2022-11-18 4:10 pm	2022-11-18 4:39 pm
	30	5–70 Hz	2022-11-18 4:41 pm	2022-11-18 5:10 pm
	30	10–70 Hz	2022-11-18 5:12 pm	2022-11-18 5:41 pm
	30	20–50 Hz	2022-11-18 5:40 pm	2022-11-18 6:12 pm
	30	20–60 Hz	2022-11-18 6:14 pm	2022-11-18 6:43 pm
	832	10–50 Hz	2022-11-18 6:45 pm	2022-11-19 8:36 am
25 m	231	10–60 Hz	2022-11-19 10:22 am	2022-11-19 2:12 pm
	1097	10–50 Hz	2022-11-19 2:15 pm	2022-11-20 8:31 am
50 m	231	10–60 Hz	2022-11-20 10:11 am	2022-11-20 2:01 pm
	9761	10–50 Hz	2022-11-20 2:04 pm	2022-11-27 8:43 am
	33	5–60 Hz	2022-11-27 9:47 am	2022-11-27 10:19 am
	33	5–70 Hz	2022-11-27 10:22 am	2022-11-27 10:54 am
	33	10–70 Hz	2022-11-27 10:57 am	2022-11-27 11:29 am
	33	20–50 Hz	2022-11-27 11:31	2022-11-27 12:03 pm
	33	20–60 Hz	2022-11-27 12:05 pm	2022-11-27 12:37 pm

## Data

**Fig. 2:**
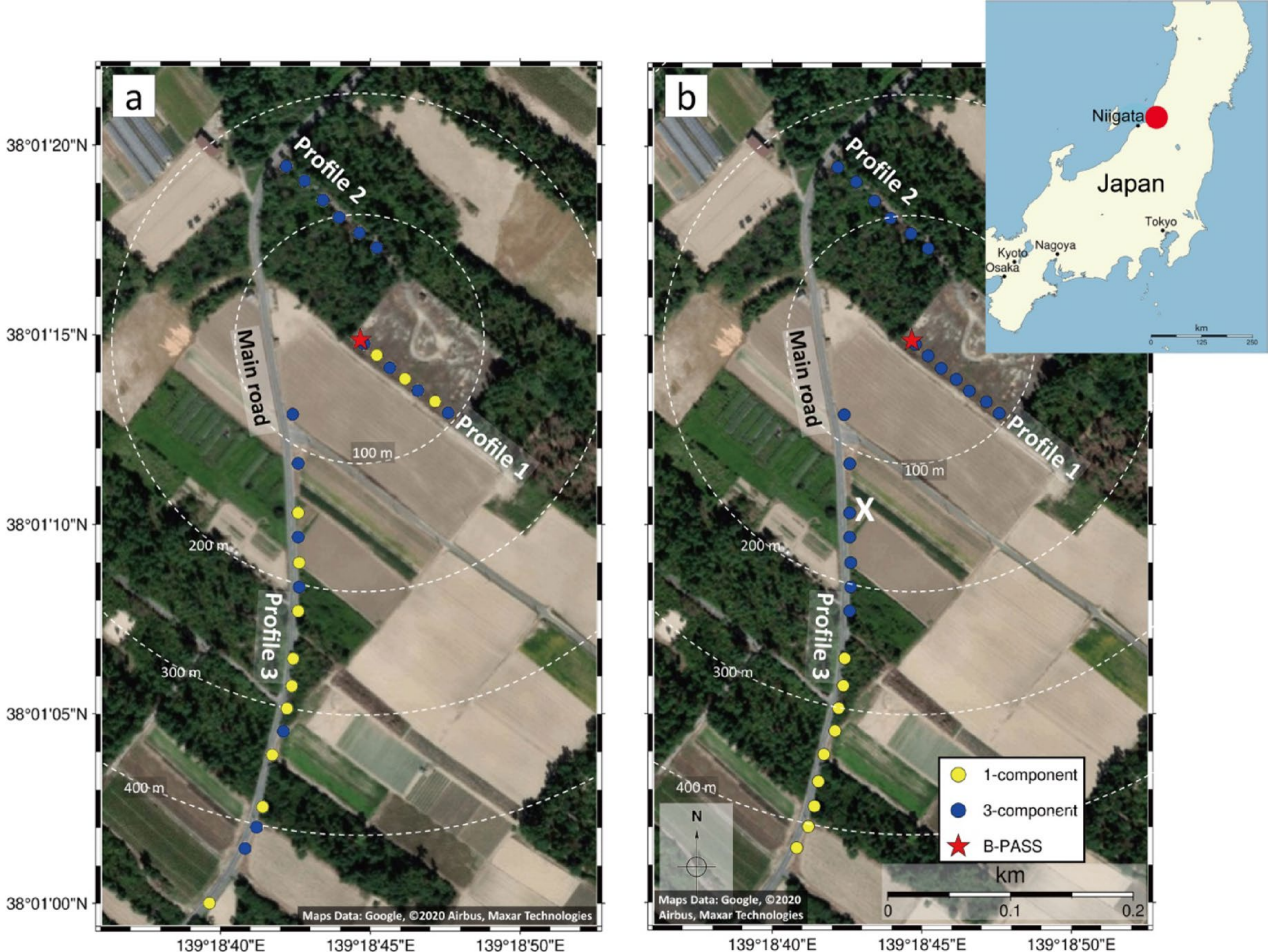
Maps of the study area of the B-PASS system experiment in the Nakajo oil field, showing the configuration of geophones (blue and yellow circles), the location of the B-PASS system (red star), and the locations of the three profiles. “X” is a station located at 150 m offset. Sensor distributions are shown for the (**a**) horizontal-motion B-PASS system configuration and (**b**) vertical system configuration. The satellite images in (**a**) and (**b**) are obtained from Google Earth Web (© Google, Image Landsat/Copernicus; Version 10.85.0.1), prepared and annotated with PyGMT (v0.14.2, https://www.pygmt.org).

Field testing of the B-PASS system was conducted at a gas field in Nakajo, Niigata, northern Japan (Fig. 2). The site was chosen due to the strong seismic attenuation in the shallow beach-sand layer, which required the seismic source to be deployed at greater depths. These geological conditions are similar to many offshore CO_2_ storage sites, making Nakajo an ideal location for testing. A 50 m deep borehole was used to evaluate the system’s performance across various frequency ranges, including: 0.5–60, 0.5–70, 10–50, 10–60, 10–70, 20–50, and 20–60 Hz, at depths of 0.5, 25, and 50 m as illustrated in Tables 1 and 2.

**Table 2:** Operation and parameter settings of the vertical-motion B-PASS system.

Source depth	Sweeps	Frequency	Start time (JST)	End time (JST)
0.5 m	2250	10–50 Hz	2023-05-17 1:52 pm	2023-05-18 8:46 am
25 m	450	10–50 Hz	2023-05-18 9:34 am	2023-05-18 2:00 pm
50 m	1045	10–50 Hz	2023-05-18 2:35 PM	2023-05-18 11:18 PM
	460	10–50 Hz	2023-05-19 6:15 am	2023-05-19 10:00 am

The experiment was conducted in two phases to assess the system. The first phase, conducted in November 2022, focused on the horizontal-motion B-PASS system. This phase involved an up-hole survey to evaluate both the distance and stability of signal propagation. Sweep signals were emitted at 30-second intervals, with 29 geophones deployed across three profiles to record data. Geophone spacing was 10 m for Profile 1, 15 m for Profile 2, and approximately 20 m for Profile 3 (Fig. 2). The maximum horizontal offset between the source and receivers was 470 m, with sensors including both 1-component and 3-component geophones. The geophones recorded seismic signals at a sampling rate of 500 Hz (2 ms). The 3-component geophones were equipped with batteries capable of lasting one month, while the 1-component geophones required recharging every two days. Battery replacements were conducted daily between 8:00 AM and 9:00 AM.

The second phase of testing, conducted in May 2023, assessed the vertical-motion B-PASS system in a similar up-hole survey lasting three days. For this phase, the 29 geophones were rearranged into new profiles, with the maximum horizontal offset reduced to 425 m. As in the first phase, the B-PASS system emitted sweep signals at 30-second intervals, but the geophones recorded data at a higher sampling rate of 1000 Hz (1 ms) for improved resolution. The arrangement of geophones and operational details were modified to explore the vertical-motion system’s performance in a different configuration, ensuring a comprehensive evaluation.

During both phases, the transfer functions of each stacked frequency band were systematically analyzed at all tested depths. Through these tests, we tried to evaluate the reliability and adaptability of the B-PASS system across a range of configurations and environmental conditions in challenging geological settings.

## Method

**Fig. 3:**
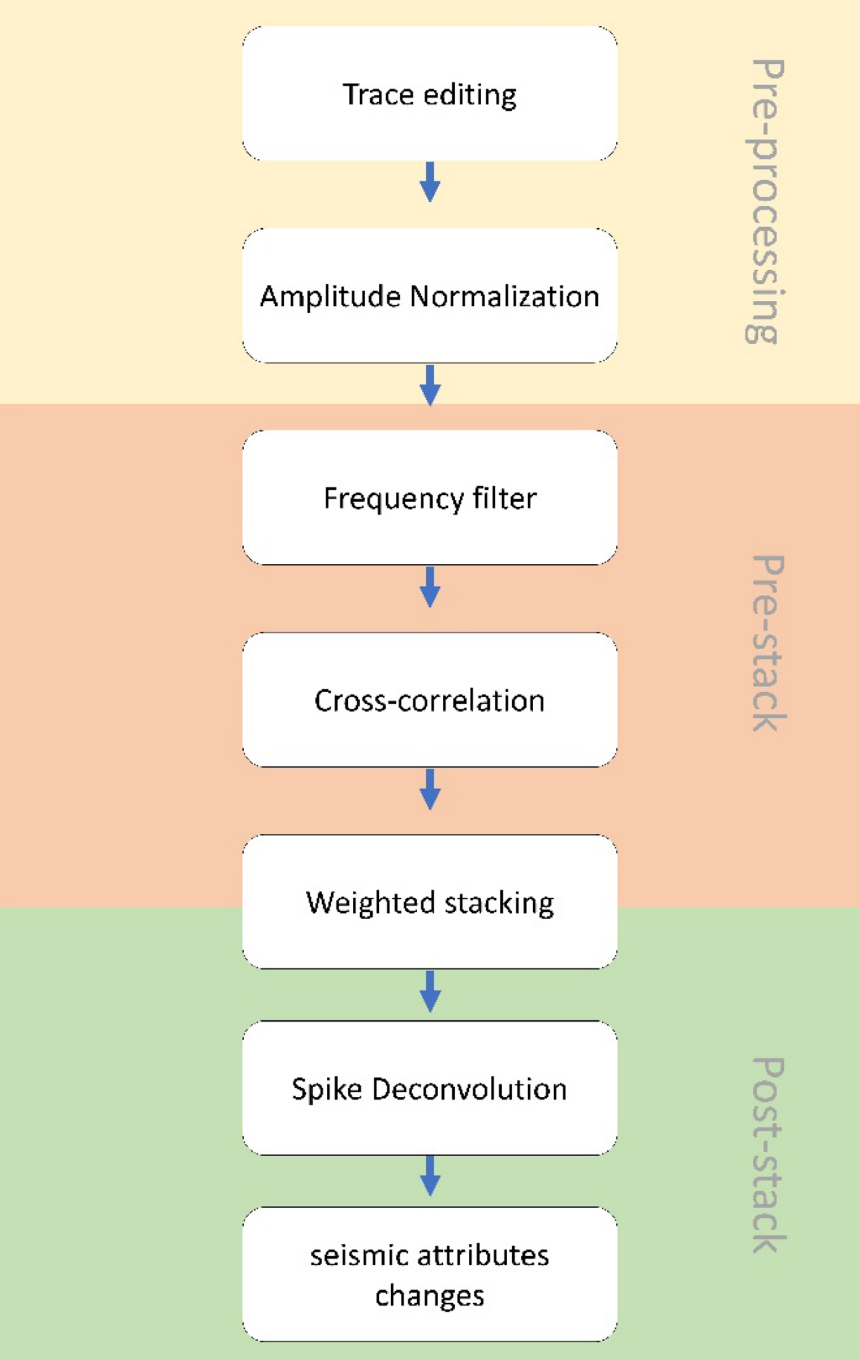
Flowchart of the key data processing steps. The flow includes pre-processing, pre-stack analysis (frequency filtering, cross-correlation), and post-stack enhancement (weighted stacking, spike deconvolution, attribute analysis).

**Fig. 4:**
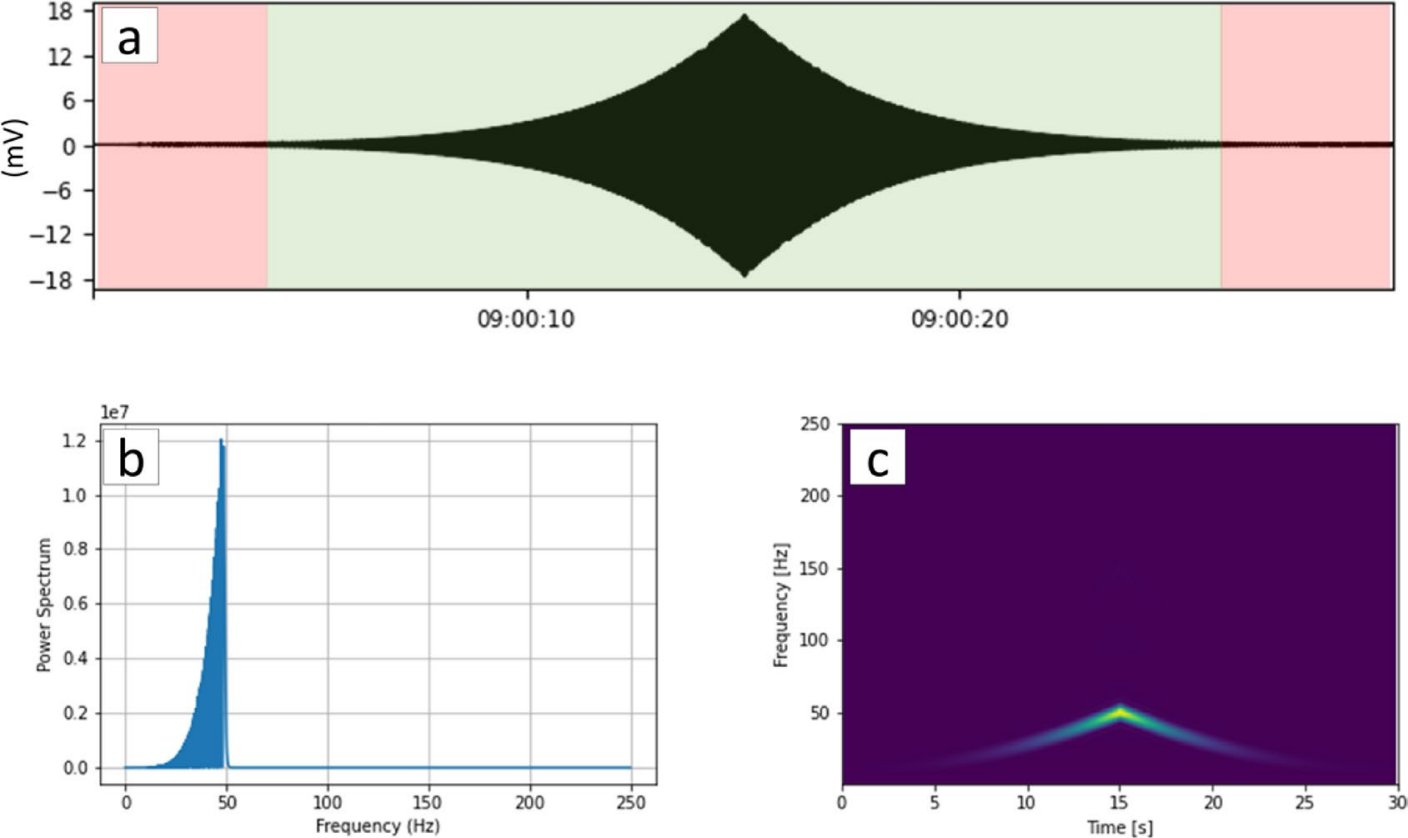
Vertical-motion B-PASS source function and its spectra at 50 m depth with a 10–50 Hz operating frequency. (**a**) An individual 30-second sweep waveform with a signal (green) and a quiet window (red) is used for S/N ratio calculation. (**b**) Power spectrum and (**c**) spectrogram related to the sweep waveform shown in (**a**).

The B-PASS system utilizes a low-amplitude waveform but is repetitive. Through stacking these precisely repeated waveforms, the system enhances the S/N ratio of the monitoring signal, consequently reducing random noise. The workflow is illustrated in Fig. 3. The first step involves the application of a bandpass filter on the receiver data, aligning with the frequency range of the B-PASS operation (Fig. 4b and c). Subsequently, a transfer function for the B-PASS is calculated using a cross-correlation method^37^:

1 $$\:x\text{*}y\left(\tau\:\right)=\:\int\limits_{-{\infty\:}}^{{\infty\:}}x\left(t\right)y\left(t+\tau\:\right)dt$$

where$\:\:x*y\left(\tau\:\right)$ represents the cross-correlation between two seismic traces *x* (source function of B-PASS) and *y* (receiver) at time t, and $\:\tau\:$ is the lag parameter, indicating the degree to which *y(t)* is shifted with respect to *x (t)*. We used a built-in B-PASS geophone for the source function (Fig. 4). In the cross-correlation analysis, a time window equivalent to the duration of each sweep (30 s) is employed.

In the next step, the cross-correlated trace of each sweep is weighted based on the S/N ratio of that sweep. This weighting process involves evaluating the variance of both the silent and sweep windows, shown in red in Fig. 4a. These variance values are used to determine the weight of each sweep and to implement a weighted stacking technique as follows:

2 $$\:G\left(k\right)\:=\:\frac{{\sum\:}_{n}\frac{\:{g}_{n}\left(k\right)}{var\left[{noise}_{n}\right]}}{{\sum\:}_{n}var[{noise}_{n}{]}^{-1}}$$

where *G(k)* is the stacking result of *n* sweeps after calculating the variance *(var)*; *g*_*n*_*(k)* is the transfer function in the time domain; *noise*_*n*_ is the window of the noise at the end of each sweep of *n*, which is an index of operation; and *k* is an index of time series data. Adopting weighted stacking, as opposed to simple average stacking, is a key part of this procedure. We carefully considered the distinct quality and noise characteristics of each sweep by employing weighted stacking. This approach leads to a more refined and accurate outcome during the stacking process, as sweeps with noisier signals or a lower S/N ratio have less effect on the overall stacking. To complete the data processing, we incorporate spiking deconvolution and enhance the resolution of seismic data^38,39^.

## Results and discussion

### Source repeatability

Seismic monitoring aims to detect subtle temporal variations of the deep reservoir conditions. The accuracy of seismic monitoring is highly dependent on the stability and consistency of the source signal. This study is the first to test a continuous source in a borehole, and any temporal fluctuations in the source signal could undermine the accuracy and reliability of the monitoring results.

**Fig. 5:**
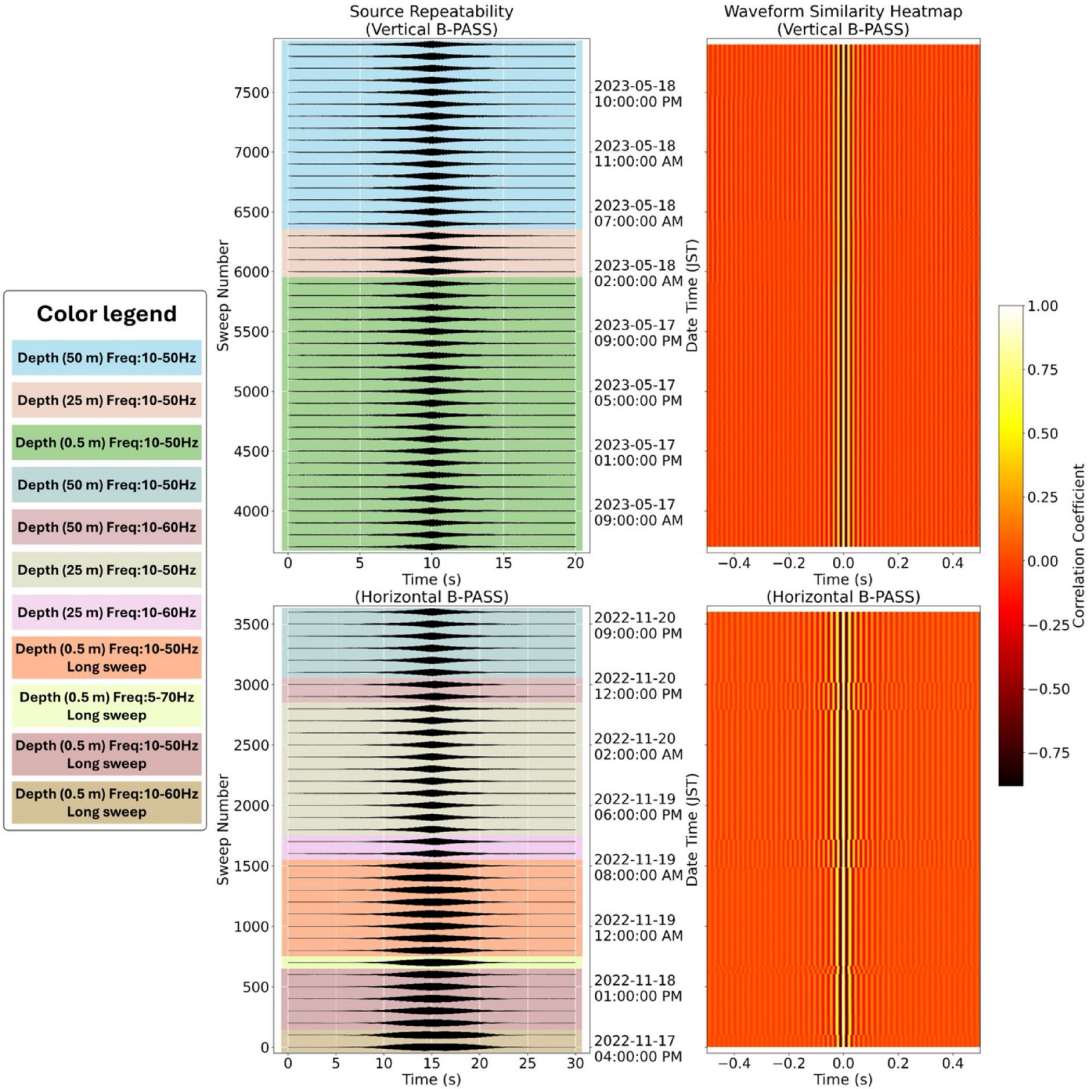
Assessment of B-PASS signal stability during a 10-day borehole deployment, evaluated over 8,000 sweeps across seven days. Left panels show the recorded waveforms for each sweep for the (top) vertical and (bottom) horizontal B-PASS configurations. Right panels present the corresponding waveform similarity heatmaps, computed as the correlation coefficient with the reference sweep.

While the source remained deployed in the borehole for 10 days, repeatability tests were conducted over nearly seven days, during which more than 8,000 sweeps were recorded using both vertical and horizontal B-PASS configurations across various depths and frequency ranges. Temporal variations in the phase and amplitude of the estimated transfer functions were minimal for each source parameter and depth (Fig. 5). Furthermore, cross-correlation analysis shows that the waveform’s shape and timing remained remarkably stable during multi-day operation. In this analysis, the first sweep at each new frequency served as the reference trace. The timestamps in Fig. 5 directly correspond to the experimental conditions detailed in Tables 1 and 2, reinforcing that the B-PASS signal is highly repeatable regardless of the operating frequency or source depth.

These results ensure that any observed changes in recorded waveforms can be attributed to true subsurface variations rather than source instability within the borehole. The high degree of similarity supports the consistency of the B-PASS system in generating stable signals by the hydraulic coupling mechanism between the source and the borehole (Figs. 1b-d).

### Signal enhancement by stacking

**Fig. 6:**
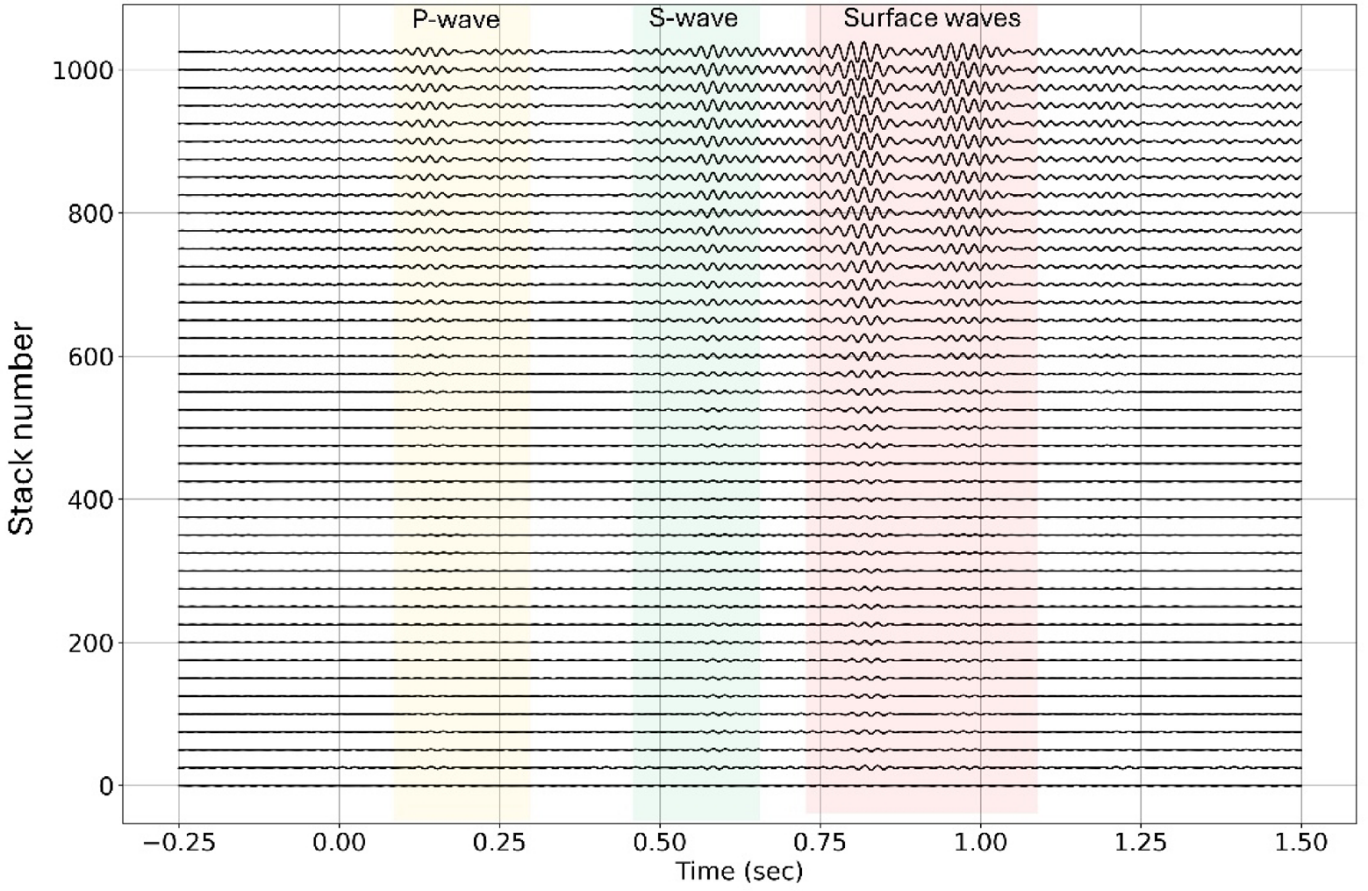
Stacked signal effect at 150 m offset. Enhanced P-, S-, and surface waves with cumulative stacking for every 25 sweeps using cross-correlations are shown.

Stacking continuous chirp signals acquired from the B-PASS system significantly enhances the signal while mitigating noise. The transfer functions established between the source and a three-component seismometer, positioned 150 m from the borehole (marked as “X” in Fig. 2b), demonstrate a notable improvement in signal quality as data from extended periods are stacked together (Fig. 6). Consequently, this stacking process ideally enables a temporal resolution of approximately 6.5 h or 800 sweeps for the seismometer positioned 150 m from the borehole. This outcome underscores the effectiveness of stacking in enhancing signal quality and extending the temporal resolution of monitoring. In Fig. 6, we can observe the first arrival of P-waves at ~ 0.1 s (~ 1600 m/s) and S-waves at ~ 0.5 s (~ 300 m/s).

The B-PASS system generates both P- and S-waves, with vertical and horizontal motions determined by the configuration of the rotational axes. To assess the impact of each B-PASS system design, we compared them using three-component seismometers in profile 1, positioned near the borehole (up to 100 m from the borehole; Fig. 2a and b). The objective was to observe how vertical and horizontal motions are manifested at the same offset. To quantify the magnitude of motion in each component, we employed the root-mean-square (RMS) for a single sweep, calculated as follows:

3 $$\:RMS=\:\sqrt{\frac{1}{n}\sum\limits_{i=1}^{n}{x}_{i}^{2}}$$

where *n* represents the number of samples and *x*_*i*_ denotes the motion amplitude for each component^40^ at seismometer “X” in profile 2 at 150 m offset (Fig. 2). A 6-hour nighttime window was selected for both vertical and horizontal B-PASS operations, with the source deployed at a depth of 50 m.

**Fig. 7:**
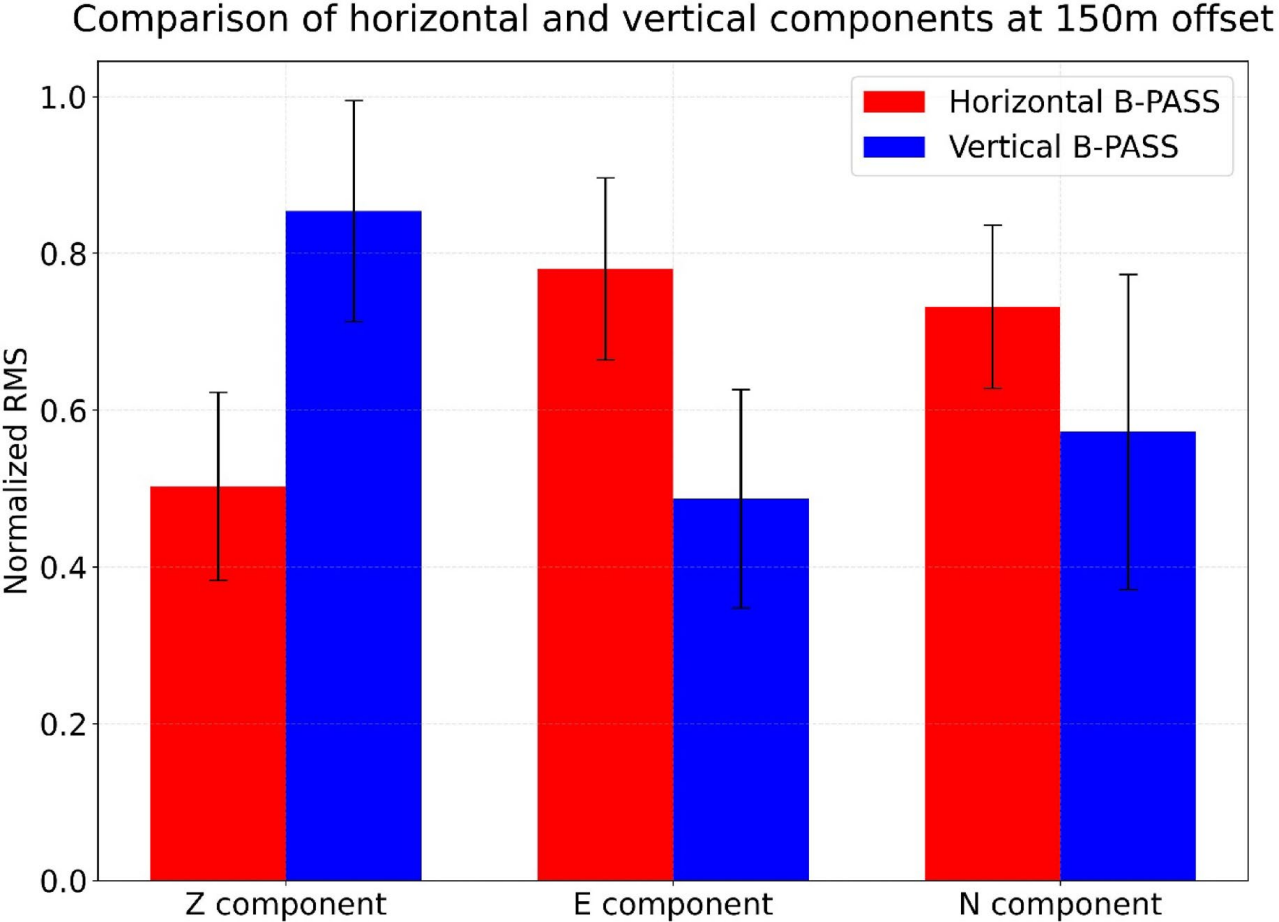
Comparison of the normalized RMS values of each motion component (E, N, and Z) between the vertical- and horizontal-motion B-PASS system design at 150 m offset (see Fig. 2 for station “X”).

Figure 7 shows the normalized RMS data with standard deviation for each component. This normalization was implemented to eliminate the offset effect for seismometers and highlights the RMS at each seismometer. For the E–W motion component, the RMS for the horizontal-motion B-PASS is 35% higher than that for the vertical-motion B-PASS. In contrast, for the vertical motion component, the RMS for the vertical-motion B-PASS is twice as high as that for the horizontal-motion B-PASS. This result indicates that the horizontal-motion B-PASS system generates more displacement on the horizontal component. In contrast, the vertical-motion B-PASS system generates greater displacement on the vertical-component. However, the horizontal and vertical motion on the N–S component was not significantly different, with the horizontal-motion B-PASS being only 5% higher than that for the vertical-motion B-PASS. This observation can be attributed to the orientation of the built-in geophone within the B-PASS system used for the source function. The geophone orientation aligns with the E-W direction, resulting in stronger vibration energy for E-W motion.

### Signal propagation distance

**Fig. 8:**
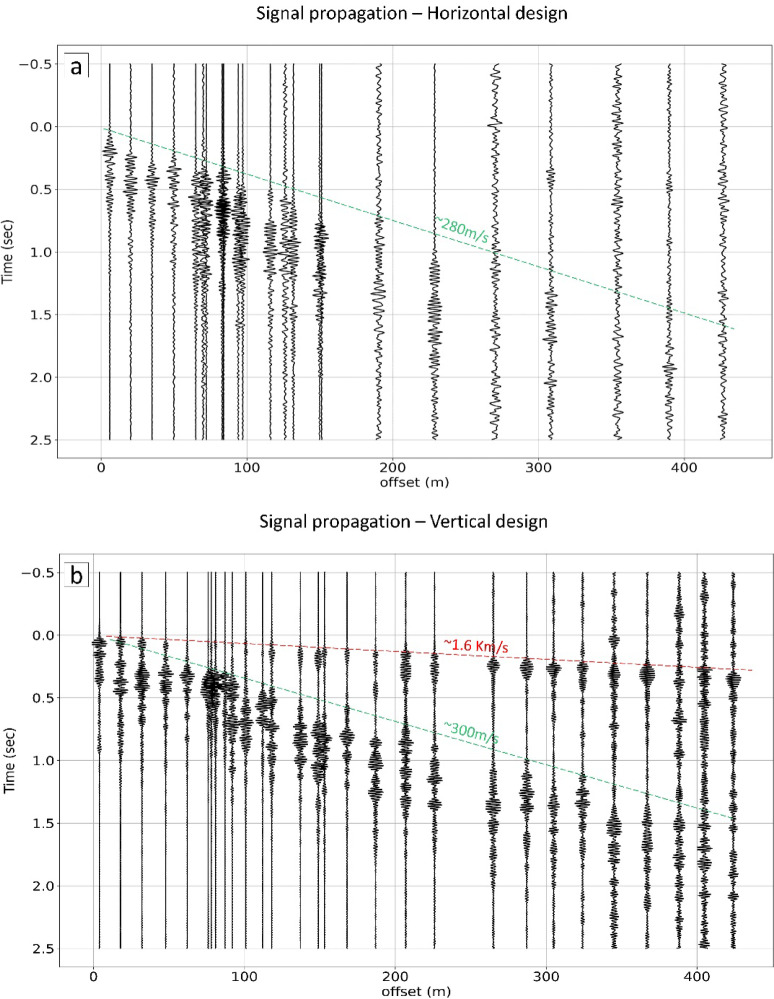
Signal propagation for (**a**) the horizontal-motion B-PASS system and (**b**) the vertical-motion B-PASS system at 50 m depth after stacking 1000 sweeps for both B-PASS systems. P- waves are denoted by red, and S- and surface waves are denoted by green.

To evaluate the extent of signal propagation generated by the PASS system underground, we conducted a careful analysis of the signals recorded by all seismometers along the three profiles (refer to Fig. 2 for the configuration). Signal propagation using the horizontal-motion B-PASS system (Fig. 8a) revealed that surface waves and S-waves propagated up to an offset of 400 m, although clear P-waves were not evident. Additionally, we examined the signal propagation for horizontal components at various frequencies using the B-PASS system.

For the signal propagation analysis using the vertical-motion B-PASS and vertical-component receivers (Fig. 8b), the initial wavefield arrival likely corresponds to the P-wave. Despite the compact design of our vertical source, we were able to discern that the body wave signal propagated over a distance exceeding 425 m (i.e., the maximum offset length). This considerable propagation distance can be attributed to the precise and continuous nature of the signals generated by our source system. The extensive propagation distances observed for both P- and S-waves demonstrate the potential of our source system for monitoring a extensive geographical area.

### Influence of source depth

We evaluated the S/N ratio of P- and S-wave to assess the impact of depth and source design of the B-PASS system. Specifically, we compared the performance of the conventional PASS^17^ placed on the surface near the wellhead with both configurations of the B-PASS system operated at three different depths. For this analysis, the vertical-component of the station at 150 m (denoted as “X” in Fig. 2b) was used for all conditions to eliminate the offset effect. The stacked data was limited to a five-hour recording window, from 8:00 PM to 1:00 AM, for each test condition. Due to data unavailability for the vertical B-PASS at a depth of 25 m during the nighttime window (i.e., 8:00 PM to 1:00 AM), we used data from a morning recording period for this specific condition.

The S/N ratio was calculated using the root mean square (RMS) method, as defined in Eq. (4), where the RMS of the signal window was divided by the RMS of the noise window:

4 $$\:SNR=\frac{{RMS}_{signal\:}}{{RMS}_{noise\:}}$$

The signal window for each wave component was defined (see Fig. 6). The P-wave window ranged from 0.1 to 0.3 s, the S-wave window from 0.5 to 0.65 s, and the surface-wave window from 0.7 to 1.1 s. The noise window was defined as the entire waveform, excluding the full signal window (0.1–1.1 s).

**Fig. 9:**
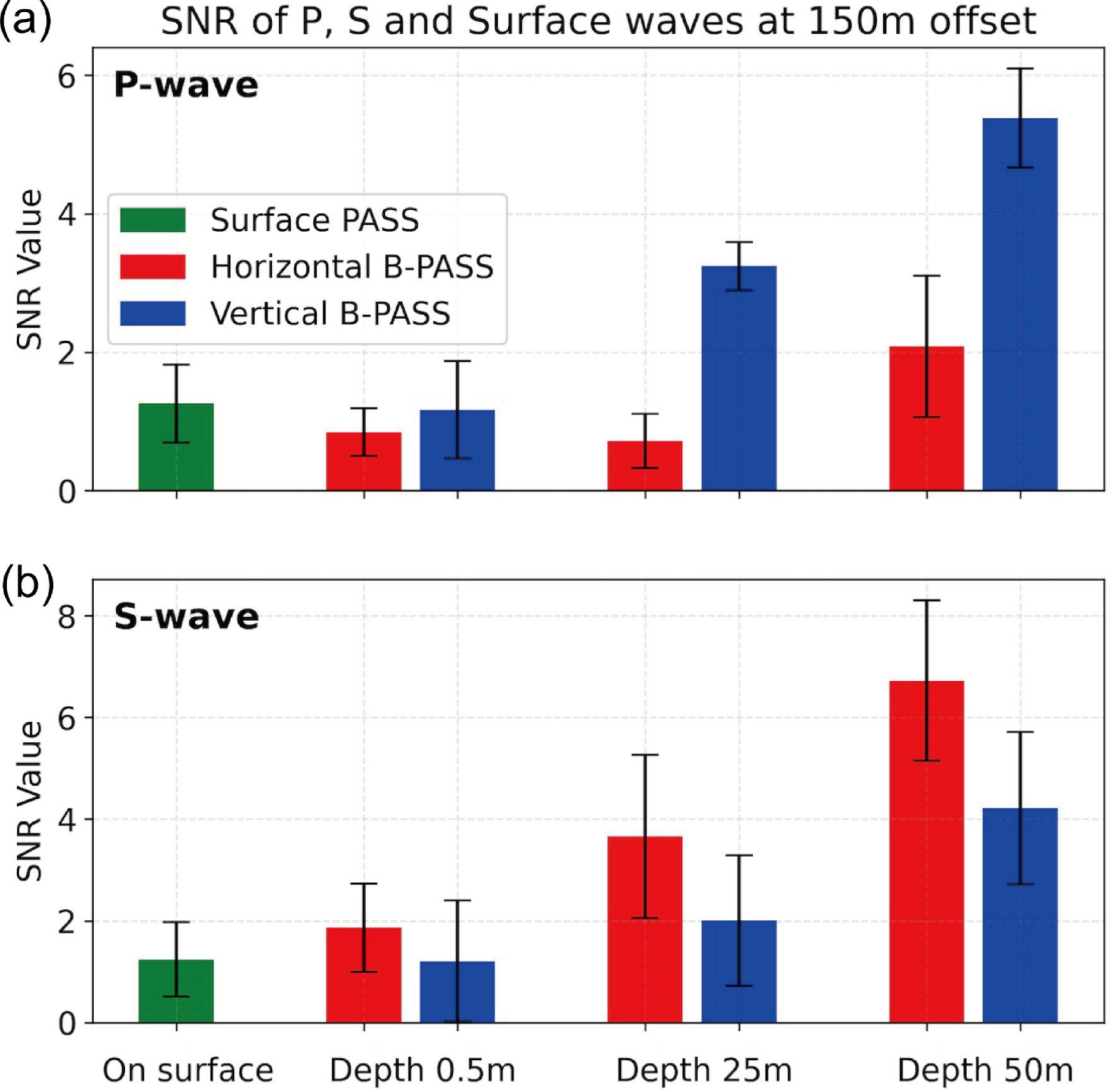
S/N ratio of (**a**) P-waves and (**b**) S-waves at 150 m offset for the Surface PASS, Horizontal B-PASS, and Vertical B-PASS systems.

The S/N ratio in Fig. 9 shows that source depth significantly improves body-wave signals. For P-waves, the surface PASS yields an S/N ratio of 1.1, while deeper sources perform better. The horizontal B-PASS at 25 m increases the S/N ratio to 3.2, and the vertical B-PASS at 50 m achieves the highest S/N ratio of 5.4, nearly five times that of the surface source (Fig. 9a). Similarly, for S-waves, the B-PASS showed better performance with a greater depth (Fig. 9b). However, unlike P-waves, the horizontal B-PASS outperformed the vertical B-PASS in terms of S-wave quality. The S/N ratio peaks at 6.0 with the horizontal B-PASS, 50% higher than the same conditions. Therefore, deeper source deployment significantly enhances the S/N ratio of P- and S-waves, with the vertical B-PASS performing best for P-waves and the horizontal B-PASS showing the highest S/N ratio for S-waves.

### Influence of environmental noise

**Fig. 10:**
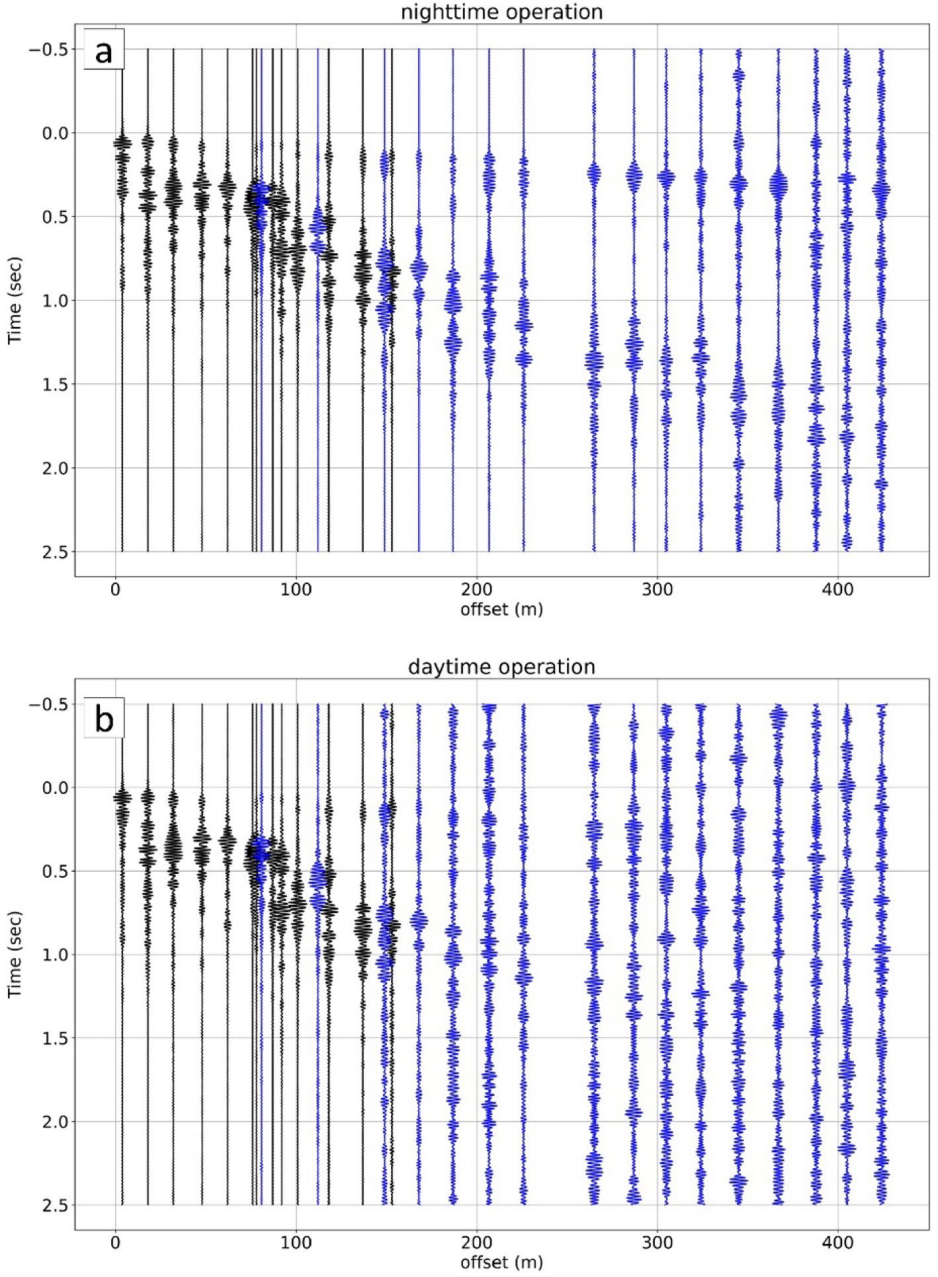
Signal propagation with 400 stacked sweeps using the vertical-motion B-PASS system. (**a**) Nighttime sweeps (6:00 PM to 9:20 PM). (**b**) Daytime sweeps (06:00 AM to 09:20 AM). Blue traces correspond to profile 3 near the main road, while black traces are far from the main road.

Considering the relatively weak nature of the signal from the PASS system, it’s crucial to account for the influence of environmental noise, including noise from human activity. This section investigates this daily temporal variation of the S/N ratio by analyzing how ambient environmental noise, primarily from human activity, impacts signal quality at different times of the day, ultimately allowing for the determination of an optimal monitoring schedule. To evaluate this influence, we operated the B-PASS system during both nighttime hours (6:40 PM to 10:00 PM) and daytime hours (6:40 AM to 10:00 AM). For this evaluation, we employed the vertical-motion B-PASS system at a depth of 50 m, applying frequencies ranging from 10 to 50 Hz. We stacked 400 sweeps (equivalent to 3.3 h) for both nighttime and daytime periods (Fig. 10). The results (i.e., shot gather or transfer function) clearly indicate much better propagation during nighttime hours.

**Fig. 11:**
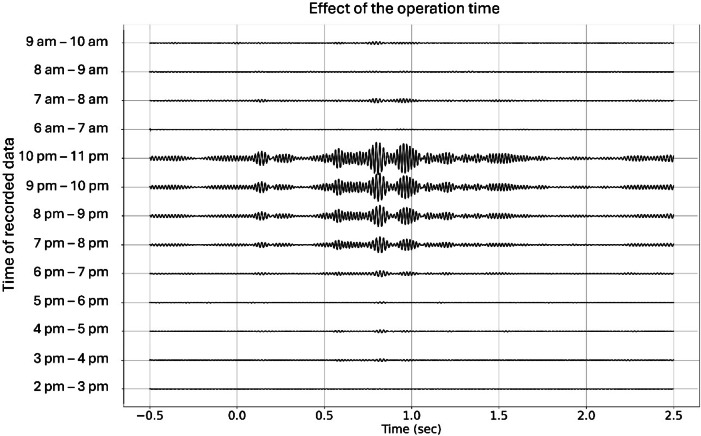
Effect of operation time on the signal at offset 150 m (marked as “X” in Fig. 2b) of the vertical-motion B-PASS system measured at a depth of 50 m. The Y-axis shows 13 operation periods: 9 periods, each lasting for 1 h (from 2 PM to 11 PM), and 4 periods, each lasting for 1 h (from 6 AM to 10 AM). Each period contains 120 stacked sweeps.

To gain a better understanding of the effect of environmental noise on the stacked signal, we calculated the transfer function between the vertical B-PASS at a depth of 50 m and geophone “X” in Fig. 2b, located on the traffic road with a 150 m offset. We stacked 120 sweeps (1 h each) for the periods of 2 PM to 11 PM and 6 AM to 10 AM, and used a band-pass filter with a range of 30–65 Hz. The resultant stacked signals close to midnight are superior to those recorded at rush hour (3–6 PM and 6–8 AM; Fig. 11), even though the number of stacked sweeps is the same in each hour. To effectively conduct the shooting operation for monitoring, the timing of the operation can be scheduled for nighttime.

**Fig. 12:**
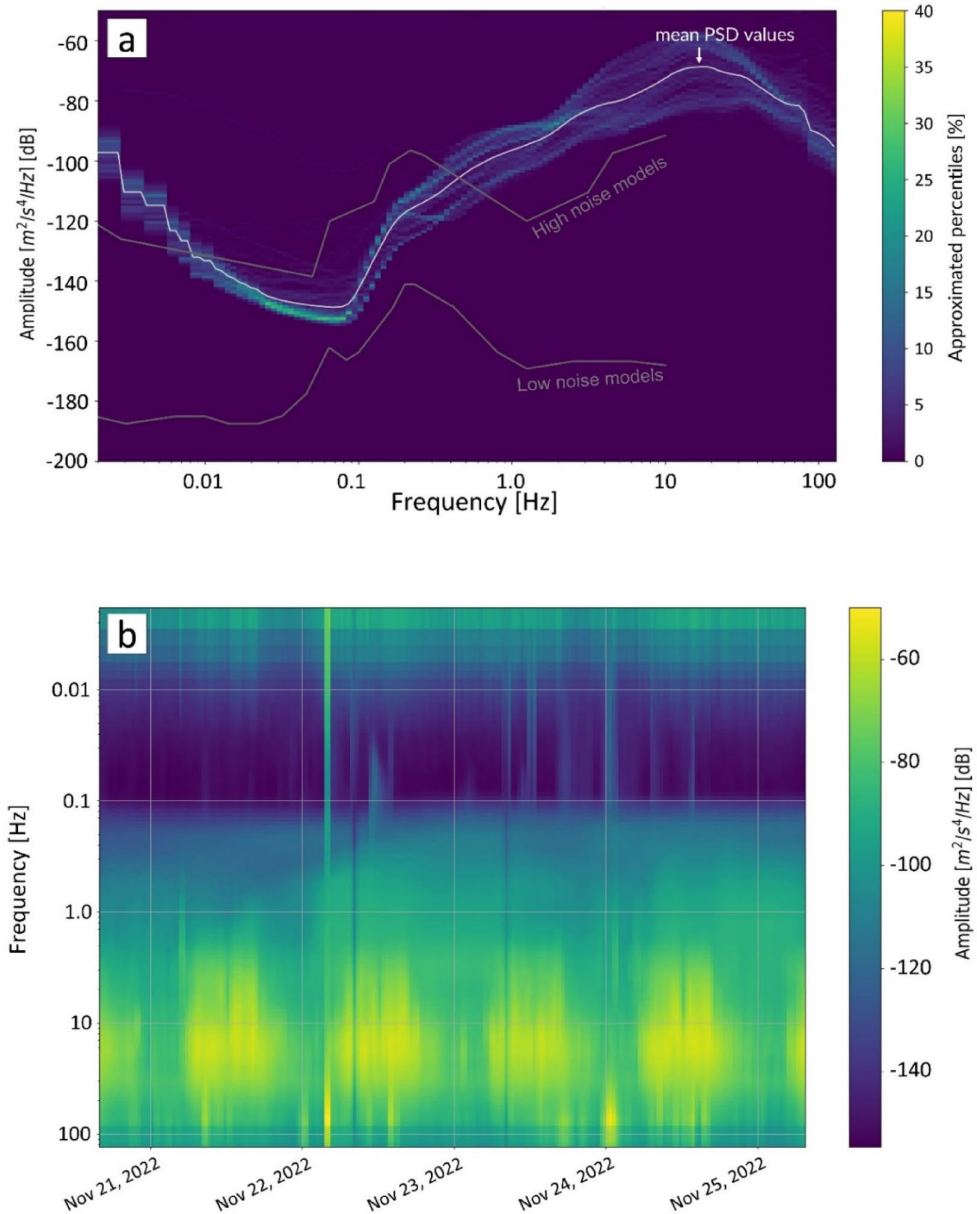
(**a**) Probability power density percentiles of approximated percentiles are calculated from the binned histogram for the profile with the mean represented by the white curve, the two gray lines represent the new high-noise model (NHNM) and new low‐noise model (NLNM)^50^ of (**b**) Spectrogram of PSD for the period 20–25 November 2022 Japan Standard Time (JST).

We calculated the probability power spectral density (PPSD^41^ to investigate the influence of noise on the results (Fig. 12). This method calculates the distribution of PSD values across frequencies and time^42^, allowing us to analyze the temporal variation in field noise characteristics. A discernible pattern is seen in the PPSD spectrogram (Fig. 12). In particular, an increase in amplitude of approximately − 70 dB emerges each day between 5 AM and 8 PM. This increased amplitude occurs across a frequency range of 10 to 100 Hz, which aligns closely with the operational range of the B-PASS system (5–70 Hz). This result (Fig. 12) suggests that human activity, mainly traffic, contributes to environmental noise in the tested location. Noise’s pronounced interference with the comparatively delicate signals from the B-PASS systems means that noise may affect the integrity of the B-PASS signals and, therefore, needs to be mitigated.

**Fig. 13:**
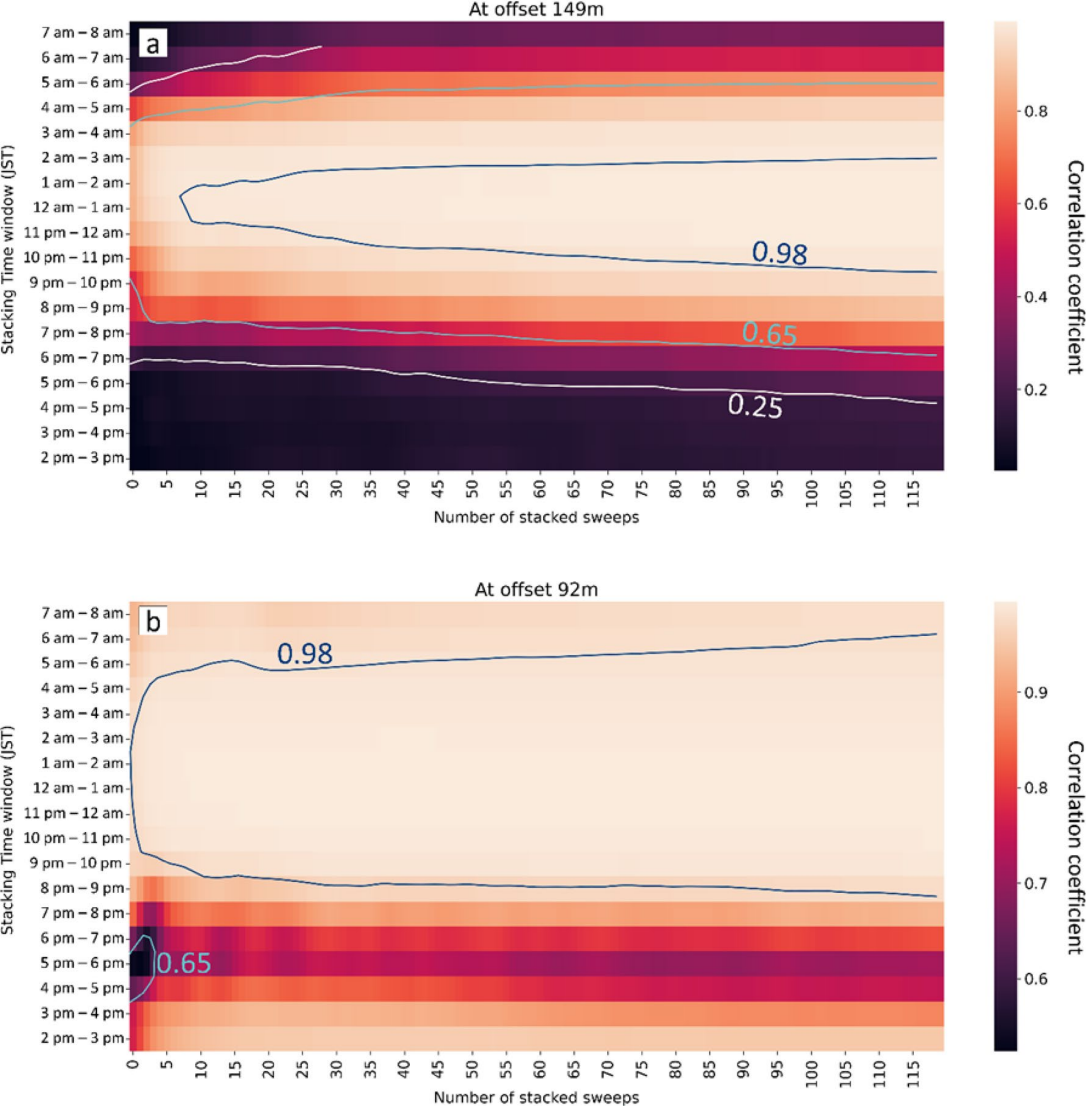
Correlation coefficient heat map between 2250 stacked sweeps and various numbers of stacked sweeps at 18 times windows from 2 PM to 8 AM the next day of the vertical-motion B-PASS system at a depth of 0.5 m (**a**) at offset 150 m (station “X”) and (**b**) at offset 92 m (farthest station at profile 1 in Fig. 2b).

To further quantify the impact of temporal variations of environmental noise, we assessed the number of sweeps needed to achieve comparable results to those obtained from a larger set of sweeps within each one-hour operational window (Fig. 13). For this analysis, we utilized continuous operation data from the period of 2 PM to 8 AM for the vertical B-PASS system at a depth of 0.5 m. We examined the stacking effect across 18 one-hour time windows, evaluating two factors: operation time and the number of sweeps. We calculated the correlation coefficient between the stacked signal accumulated at each time window and the accurate signal derived by stacking a total of 2250 shots in order to measure how many shots we needed to achieve a similar result of 2250 stacked sweeps at different times. The heat map shown in Fig. 13a demonstrates significant variations in the correlation coefficient between nighttime and daytime at offset 150 m (marked as “X” in Fig. 2b). The 10 to 20 sweeps (shots) at midnight time (11 PM to 3 AM) can generate a result 98% similar to the one generated by stacking 2250 sweeps. In contrast, even 120 sweeps of daytime periods (2 PM to 6 PM and 6 AM to 8 AM) failed to produce a stacked signal with similarity exceeding 50% at any point.

Even when we compare the results for at offset 90 m (the farthest station in profile 1 in Fig. 2b) in Fig. 13b, where the geophone is located inside the testing facility away from the main road, the influence of rush hour (3:30 PM to 7 PM) is evident, causing the correlation coefficient to drop from 95% to 80%. In other words, quiet time with less human activities, especially from 11 PM to 3 AM, can be the best time to operate the B-PASS monitoring system in this field. We observed a similar trend at all stations; all heat maps for this investigation are presented in the supplementary material (Figures S1-S3). The findings from Figs. 10, 11, 12 and 13 emphasize the critical role of temporally varying environmental noise in obtaining accurate and effective monitoring data. It is crucial to consider the timing of operations to avoid resource wastage, such as excessive electric power consumption, and mitigate the risk of mechanical failures attributable to B-PASS system operations.

### Optimum stacked number for each source-receiver distance

To optimize the temporal resolution of a monitoring system, it is necessary to determine the number of stacked sweeps required based on the offset distance between source and receivers. While using fewer stacked sweeps improves temporal resolution, it may result in a lower S/N ratio. Therefore, the goal is to determine the minimum number of sweeps required to produce a clearly recorded signal from the B-PASS system. For this purpose, we examined the stacked signal from the vertical-motion B-PASS system at a depth of 50 m, using 1025 sweeps (from 2 PM to 11 PM) as the optimal stacking reference. We then correlated this result with signals stacked cumulatively every 25 sweeps.

**Fig. 14:**
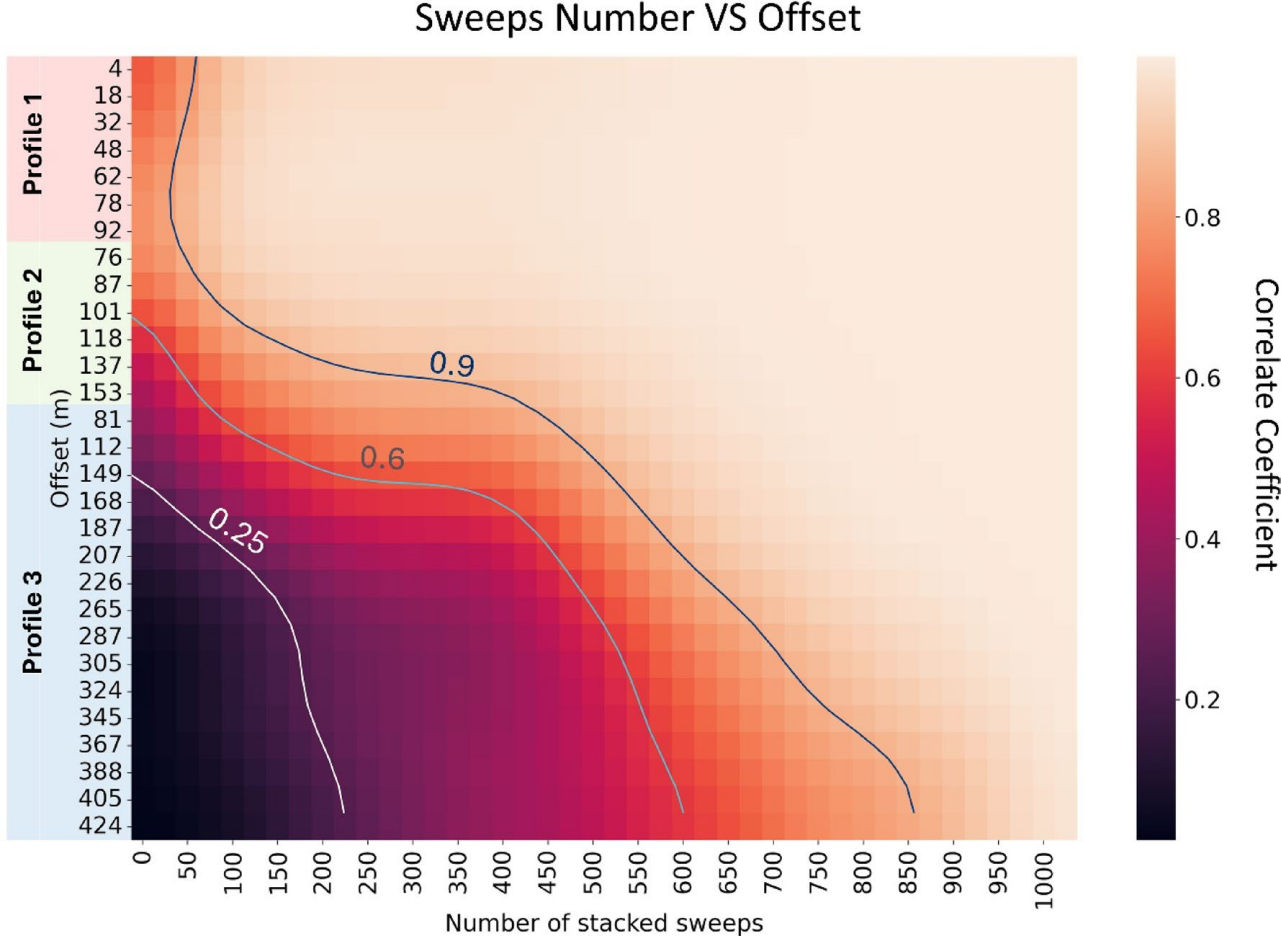
Relationship between offset distance and the number of stacked sweeps using correlation coefficients for 1025 stacked sweeps for all geophones of the vertical-motion B-PASS system at a depth of 50 m and an operation time from 2 PM to 11 PM.

The correlation coefficients indicating the S/N ratio are presented as a heatmap in Fig. 14. After only 50 stacked sweeps, profile 1 (with offset distance of less than 100 m) achieved a 90% correlation with the reference result from 1000 stacked sweeps, suggesting that 50 sweeps could be sufficient for stacking. In contrast, a greater number of sweeps is required to achieve 90% correlation at larger offsets (Fig. 14). A larger number of sweeps is needed for profile 3, which is located along a traffic road. The relationship between the number of stacked sweeps and the offset reveals that for the field conditions during the evaluation, at least 400 sweeps (3.3 h) are required to obtain a stacked signal with a good S/N ratio, with 25 additional sweeps being needed for each extra 20 m offset. The number of sweeps could vary depending on the field conditions. This evaluation scheme can be utilized in the initial phase of the monitoring, in order to fix the number of stacked sweeps for monitoring operation. Furthermore, when we found that environmental noise is too strong to obtain high S/N ratio data, the receivers can be deployed far from noise source by checking the relationship shown in Fig. 14.

### Numerical simulation for the source depth effect

**Fig. 15:**
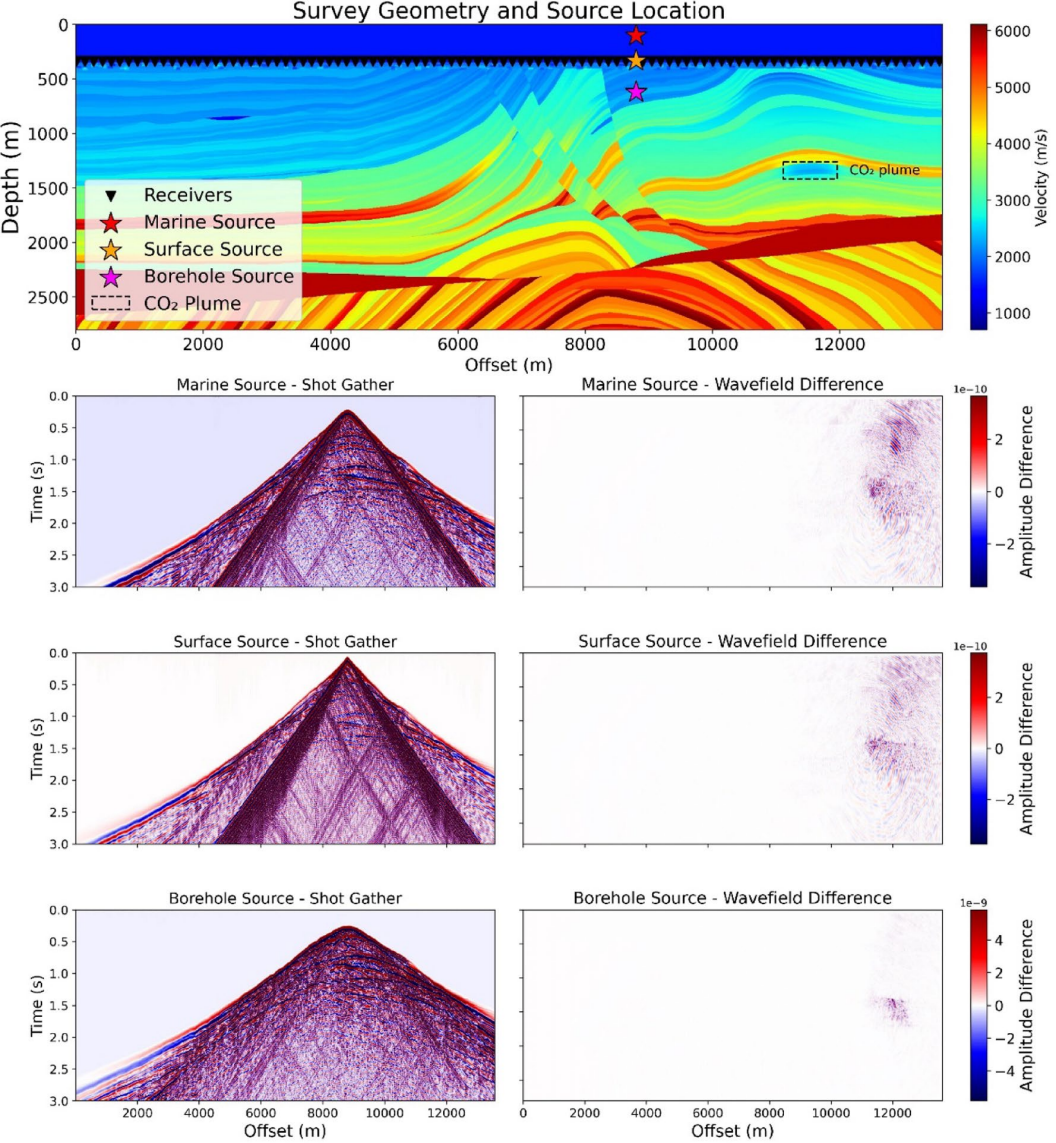
Elastic wave propagation simulations generating shot gathers and corresponding time-lapse data, comparing different source depths for deep CO₂ monitoring. Top: survey geometry over the Marmousi model with marine, surface, and borehole sources, targeting a CO₂ plume beneath a high-attenuation (scattering) surface layer. Bottom: shot gathers (left) and time-lapse wavefields (right) for each source, illustrating signal propagation and CO_2_ detectability, respectively.

To evaluate the effectiveness of the borehole source compared to the surface source in the monitoring of stored CO_2_ in a deep reservoir, we conducted an elastic wave propagation simulation using the complex Marmousi model^43^. We adapted the model to represent a challenging offshore scenario with a 350 m water column and a highly scattering (attenuation) shallow layer at depth 350–450 m (top panel of Fig. 15; see Supplementary Material for model and simulation parameters; Figure S4-S7). The simulation aimed to assess the effect of source placement on seismic signal strength for monitoring a CO_2_ plume at ~ 1,300 m depth. We tested three source configurations: a marine source (in water), a surface source (on the seafloor), and a borehole source positioned just below the attenuating (intensive scattering) layer.

For each source, we simulated shot gathers. Furthermore, we calculated the differences between shot gather before and after the CO_2_ injection, to evaluate the time-lapse response to CO_2_ injection on shot gather domain (Fig. 15). The simulations from surface and marine sources show significant noise due to surface wave and energy decreasing due to the soft shallow sediments (i.e., scattering layer). By installing the seismic source within the borehole, surface wave is much reduced and enhanced the monitoring signal derived from the injected CO_2_. This indicates that surface environmental conditions (stiffness of the shallow formation due to ice^44^) can be reduced. The coherent signals related to the CO₂ plume are observed more clearly in the borehole-source simulation, demonstrating its superior sensitivity and reliability for monitoring.

**Fig. 16:**
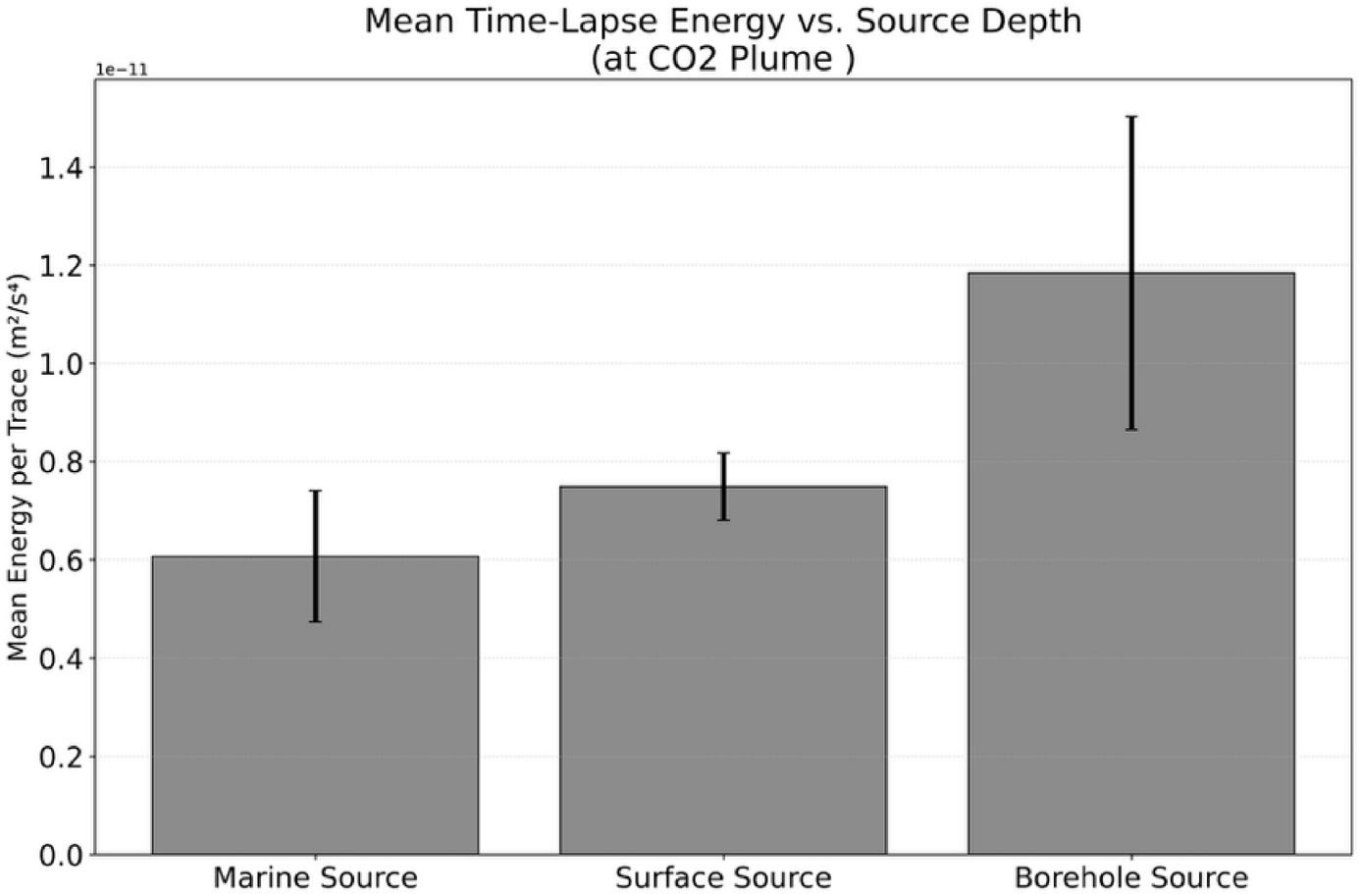
Variation in observed energy as a function of source depth. The mean time-lapse energy recorded near the CO₂ plume increases with greater source depth, emphasizing the advantage of positioning the source below the near-surface layer.

To quantify the improvement, we calculated the mean energy per trace of the time-lapse signal of the CO_2_ plume. Although the result is influenced by the model parameters, the borehole source (650 m depth) yielded a mean energy of 1.6 times higher than the surface source (350 m depth), and ~ 2 times higher than the marine source (100 m depth) (Fig. 16). This numerical results clarifys that placing a seismic source beneath shallow attenuating layers enhances the detection of CO_2_ leakage.

## Conclusion and future implications

This study optimizes the use of borehole-deployable PASS for CO_2_ monitoring by addressing limitations of the conventional surface source system and recommending optimal times and conditions for continuous monitoring surveys. Field tests verify the effectiveness of the B-PASS system in terms of signal propagation, repeatability, the impact of source depth on S/N ratio, and noise-influence mitigation. The stacking of multiple shots enables the signal to be observed up to maximum offset distance (i.e., 425 m) with 4 h of stacking.

The workflow to optimize processing and analysis includes filtering, weight stacking, and spike deconvolution to enhance the clarity of recorded signals. The source was deployed in the borehole for 10 days, during which we tested its reliability over more than 8,000 sweeps, with the source functions analyzed using cross-correlation. The sources demonstrate the system’s ability to generate stable signals consistently. We also evaluated the influence of environmental noise, such as traffic, on the recorded signals. On this basis, we propose avoiding operation of the monitoring system during daytime hours and limiting it to midnight for high S/N ratio results, and positioning the system to mitigate environmental noise to obtain better results. The S/N ratio results indicate that increasing source depth improves body-wave signals. The vertical B-PASS at 50 m achieved the highest S/N ratio for P-waves, nearly five times higher than the surface PASS, while the horizontal B-PASS outperformed the vertical B-PASS for S-waves.

The B-PASS system has the potential to be used as a seismic source for offshore environmental monitoring because we can install it inside the offshore borehole and enable strong coupling even at soft seafloor sediment. The extension to offshore scenarios remains a target for future experimental validation. Although the potential of the borehole source has been supported by the numerical simulations and technical arguments presented, direct experimental validation in a marine setting remains essential, particularly to evaluate the system’s capability to generate repeatable signals with a high signal-to-noise ratio. Seismic noise in marine environments occurs mainly at frequencies of 1 to 10 Hz^45^, which can be filtered out by data processing without affecting the signal generated by the B-PASS system. The ability of the B-PASS system to mitigate interference by surface noise and minimize damage to borehole casings makes it well-suited for long-term offshore operation. However, we should address several challenges when operating B-PASS in offshore environments. First, the PASS system uses a GPS clock to stack sweeps precisely, but GPS signals are inaccessible beneath the seawater^46,47^. Therefore, offshore versions of the B-PASS will require an alternative, high-precision timing system. Potential solutions include transmitting a timing signal directly from the surface to the downhole tool via a fiber optic cable. Second, a method for providing continuous electrical power to the downhole tool must be engineered for long-term deployments. Potential solutions range from a direct power cable connected to a surface platform or subsea infrastructure to a standalone system using a high-capacity subsea battery pack that can be periodically replaced^48,49^. Despite these challenges, the B-PASS system has the potential to revolutionize offshore monitoring by providing a reliable and cost-effective solution for seismic data collection in these challenging environments.

## Supplementary Information

Below is the link to the electronic supplementary material.

Supplementary Material 1.

## Data Availability

Original and stacked B-PASS source functions, along with formatted and pre-processed vertical seismometer datasets (including metadata) from both vertical and horizontal B-PASS experiments, are archived on Zenodo under [DOI: 10.5281/zenodo.17075241]. The seismometer data are owned by Tsuji Lab and JX Nippon Oil & Gas Exploration and may be shared upon request with approval from the data owners. Requests should be directed to the corresponding author and must include a brief analysis plan; a non-disclosure agreement may be required. All scripts used in this study to generate (Figs. 5, 6, 7, 8, 9, 10, 11, 12, 13, 14, 15 and 16) —including ObsPy/RF-Python pipelines for filtering, cross-correlation, weighted stacking, and deconvolution functions, as well as parameter and model files for the elastic-wave simulations—are openly available on GitHub at [github/AhmadBahaa1995/B-PASS.git] and archived on Zenodo under [DOI: 10.5281/zenodo.17075959].
