# Supplementary material for: Mitigating surface noise and attenuation in continuous monitoring of geologically stored CO2 using a borehole portable active seismic source
Source: Sci Rep. 2025 Oct 14;15:35856. doi: 10.1038/s41598-025-19824-8 (PMC12521397; doi:10.1038/s41598-025-19824-8)
Supplement: Supplementary file 1 — Supplementary Material 1. [file 41598_2025_19824_MOESM1_ESM.pdf]

1. Effect of urban noise on seismic signals

This section visualizes the results of heat maps to quantify the impact of temporal variations of environmental noise on vertical B-PASS at depth 50 cm in the station at 3 profiles in the Figure 3b:

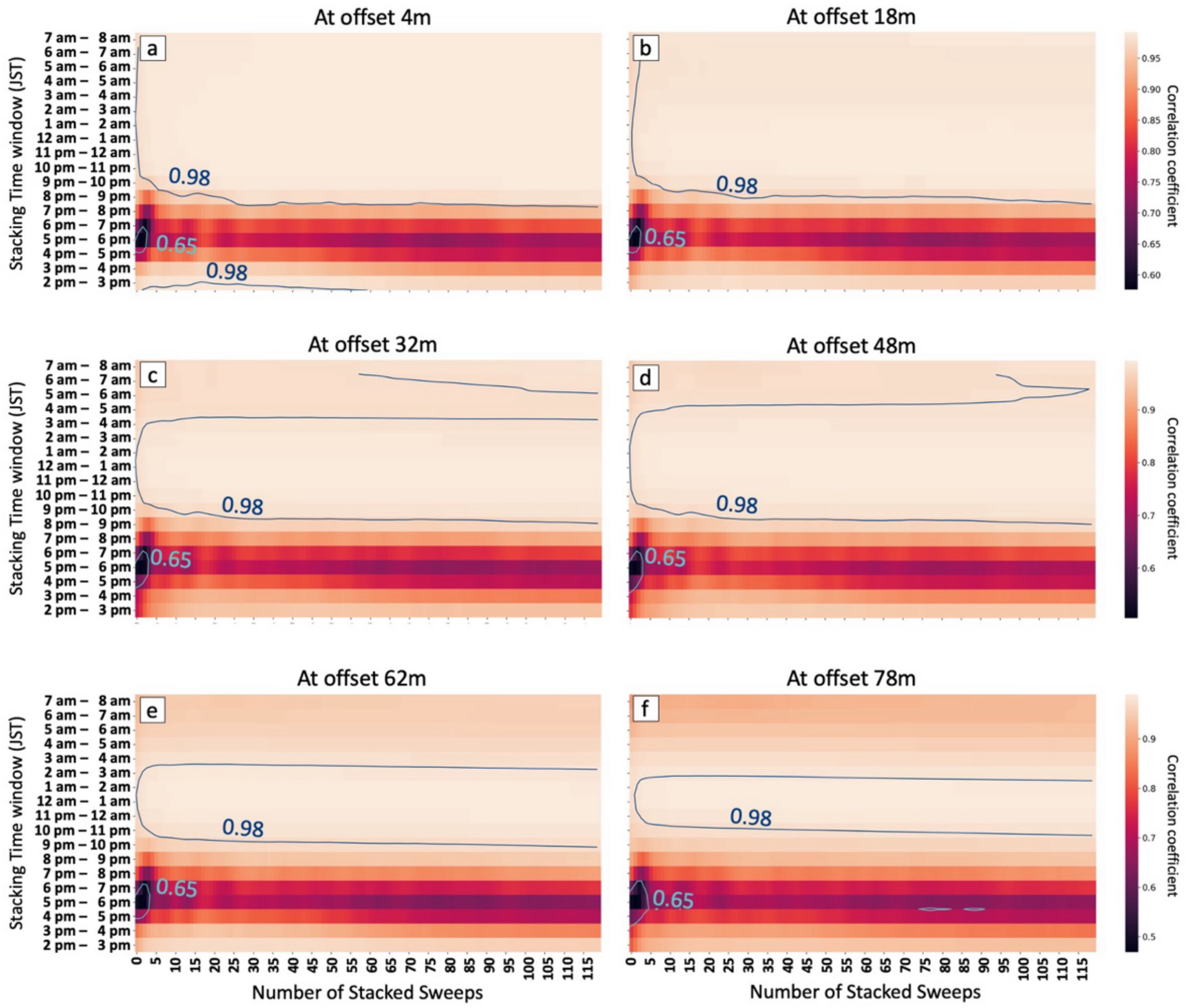

Figure S1. Correlation coefficient heat map between 2250 stacked sweeps and various numbers of stacked sweeps of the vertical-motion B-PASS system at a depth of 0.5 m for profile 1. This figure displays the correlation coefficient at 18 times windows from 2 pm to 8 am. Profile 1 includes 7stations at 4, 18, 32, 48, 62, and 78 m (panels a-f).

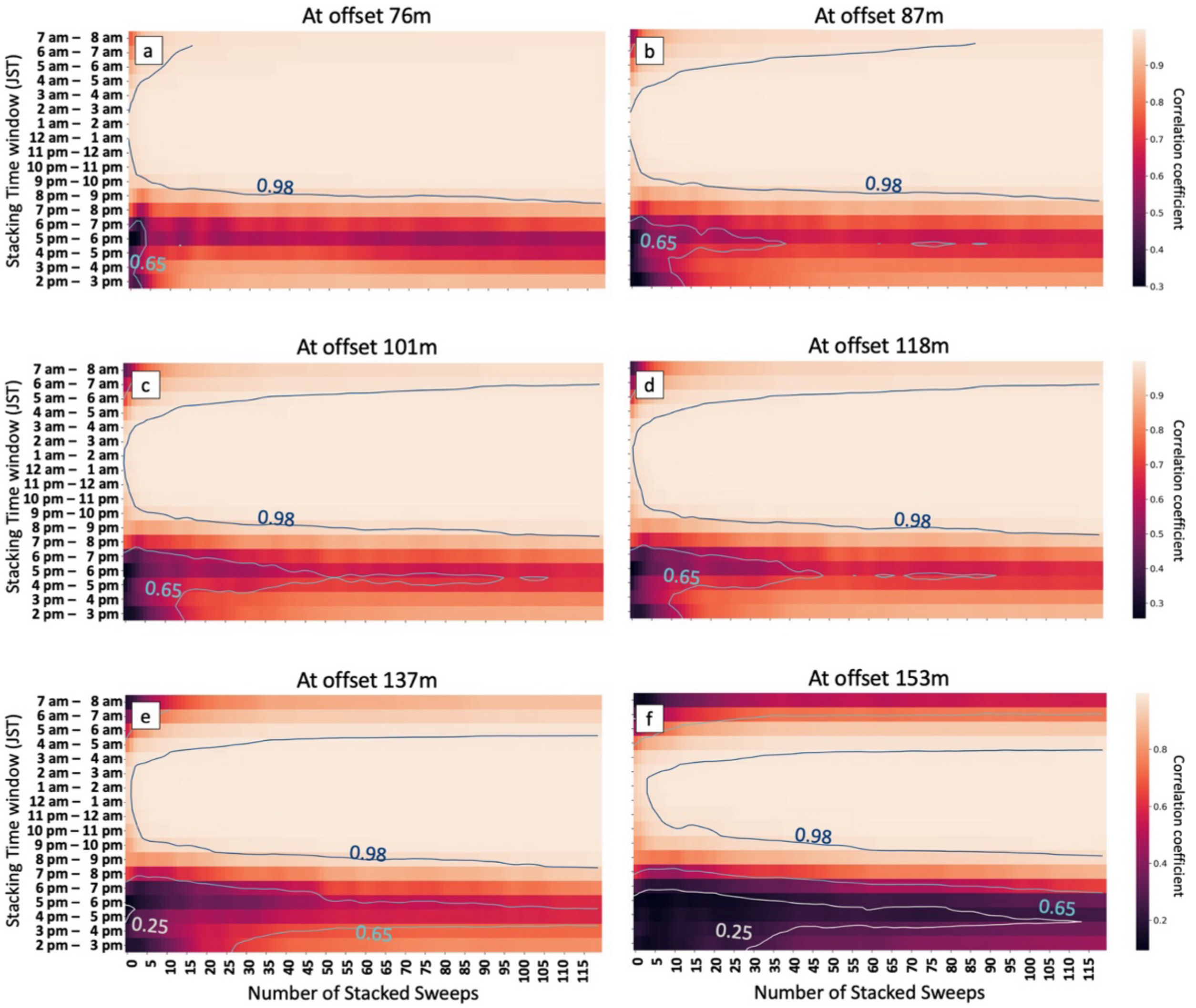

Figure S2. Correlation coefficient heat map between 2250 stacked sweeps and various numbers of stacked sweeps of the vertical-motion B-PASS system at a depth of 0.5 m for profile 2. This figure displays the correlation coefficient at 18 times windows from 2 pm to 8 am. Profile 2 includes 6 stations at 76, 87, 101, 118, 137 and 153 m (panels a-f).

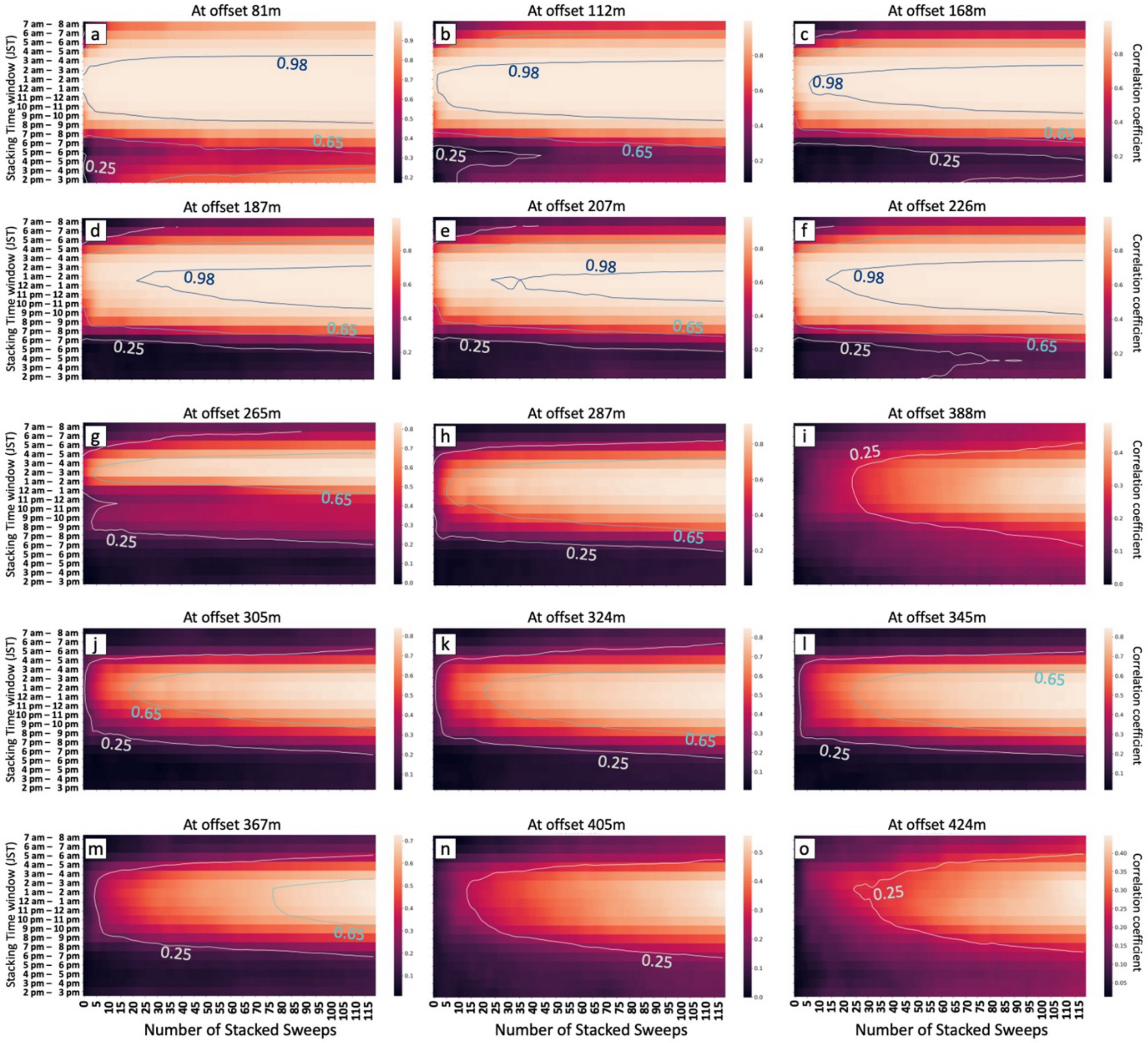

Figure S3. Correlation coefficient heat map between 2250 stacked sweeps and various numbers of stacked sweeps of the vertical-motion B-PASS system at a depth of 0.5 m for profile 3. This figure displays the correlation coefficient at 18 times windows from 2 pm to 8 am. Profile 3 includes 16 stations at 81, 112, 168, 187, 207, 226, 265, 287, 305, 324, 345, 367, 388, 405 and 424 m offset (panels a-o).

2. Numerical simulation for the source depth effect

The 2D elastic model used in this study was constructed by modifying the Marmousi synthetic benchmark. Initially, the original P-wave velocity (Vp), S-wave velocity (Vs), and density (ρ) fields were uniformly scaled by a factor of 1.3. To simulate a marine seismic acquisition environment, the shallow section was then replaced. A 350 m deep water column with constant properties (Vp=1500 m/s, Vs=0 m/s, ρ=1000 kg/m3) was placed at the top of the model. Beneath this, a 100 m thick heterogeneous sediment layer was inserted between 350 m and 450 m depth, featuring low average velocities (Vp≈850 m/s, Vs≈280 m/s) and containing small-scale random perturbations. This property at shallow formation reduce the seismic energy (causing attenuation). The deeper part of the model preserves the complex geology of Marmousi, including its characteristic folding and fault systems. The complete model, which spans 13.4 km horizontally and 2.8 km vertically, is discretized on a 4.0 m grid. A visual representation of these P-wave velocity, S-wave velocity, and density fields is provided in Figure S4.

To simulate a 4D time-lapse seismic monitoring scenario, a CO2 injection event was modeled within the baseline elastic properties. The injection targets a reservoir layer at a depth of ~1340 m and a horizontal position of 11,530 m in our model. The CO2 plume is defined with a vertical thickness of 160 m and a horizontal width of 840 m. The presence of CO2 in the pore space alters the rock's elastic properties, which were simulated by applying the following changes:

- A 25% reduction in P-wave velocity (Vp).
- A 2% increase in S-wave velocity (Vs).
- A 5% reduction in density (ρ).

To avoid artificially sharp boundaries and create a more geologically plausible plume with diffuse edges, these changes were applied using a smooth mask created by a Gaussian filter. The original model, the modified model containing the CO2 plume, and the resulting differences in elastic properties are all visualized.

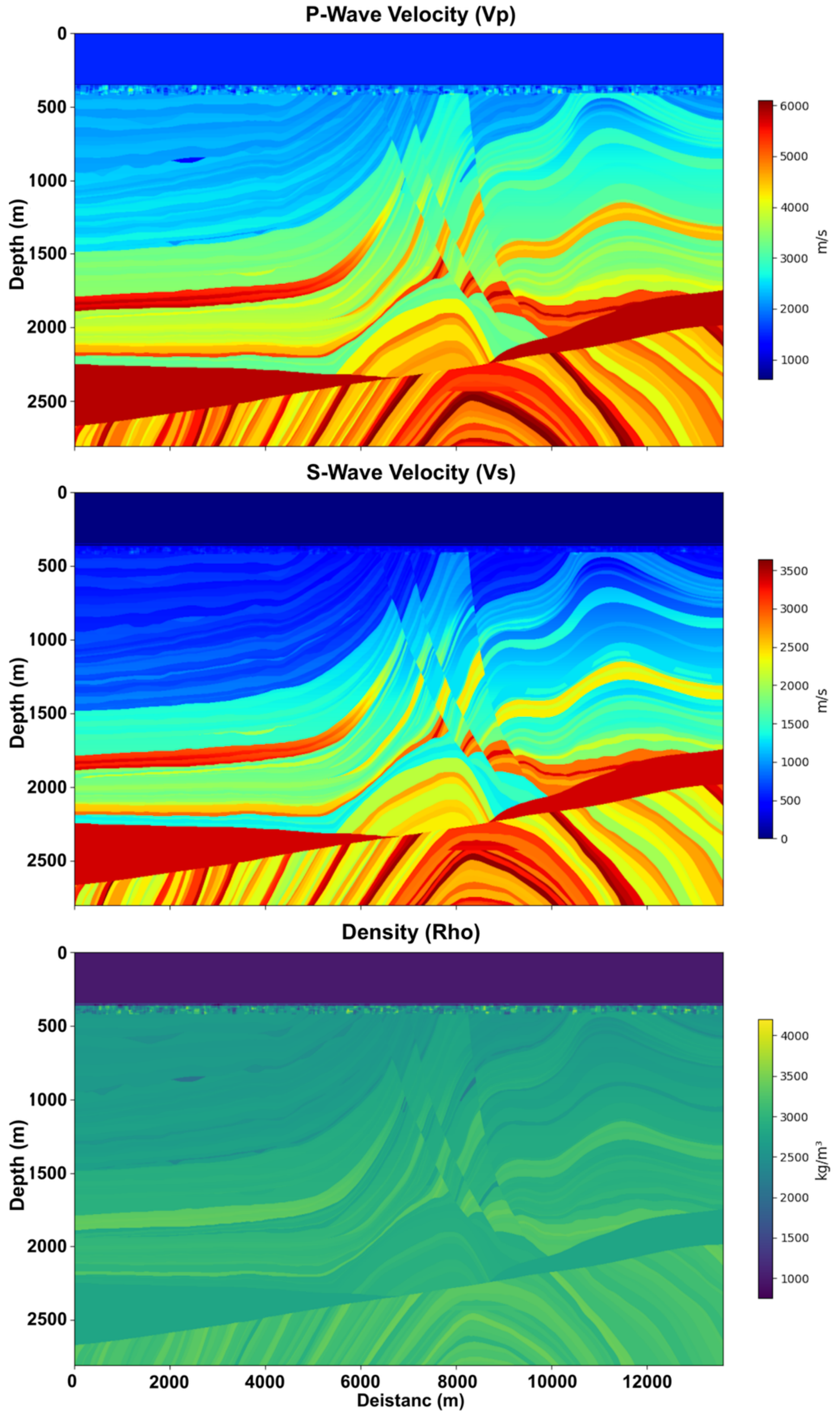

Figure S4. The 2D elastic property model used for numerical simulations, showing (top to bottom) P-wave velocity (Vp), S-wave velocity (Vs), and density (ρ). The model is a modified version of the Marmousi model which includes a water and soft sediment layer at the top.

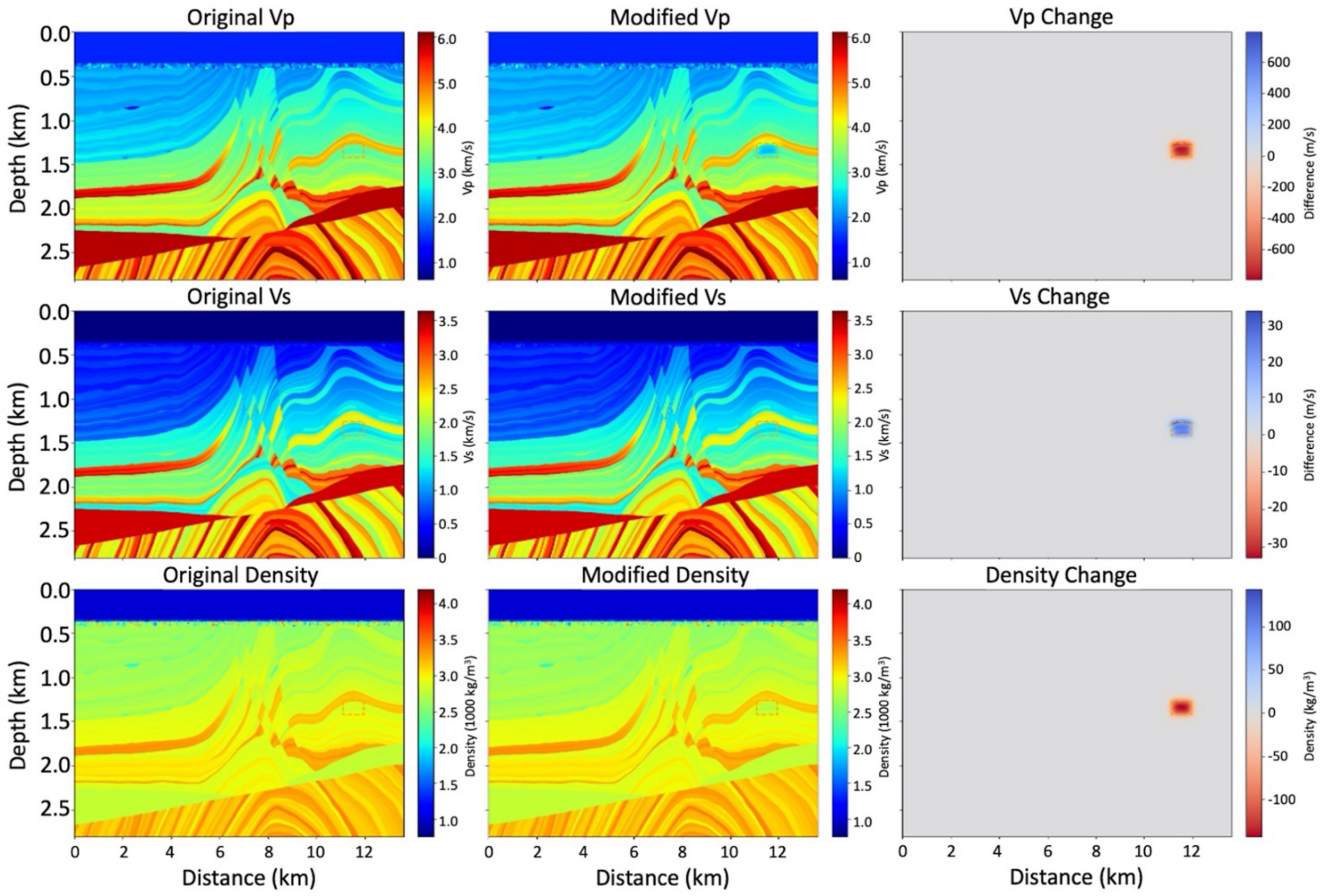

Figure S5. The effect of simulated CO2 injection on the elastic model. The rows correspond to P-wave velocity (Vp), S-wave velocity (Vs), and density (ρ). The columns show the original model (left), the model after CO2 injection (middle), and the absolute difference between them (right). The CO2 plume, centered at a depth of 1340 m, results in a significant decrease in Vp and ρ, and a slight increase in Vs. The dashed orange rectangle indicates the target injection boundary before Gaussian smoothing was applied.

To assess the time-lapse seismic response to CO_2 injection, three distinct acquisition scenarios were simulated using the DeepWave elastic wave modeling package. The goal is to compare how effectively a source at different depths can illuminate the plume. The three scenarios are:

1. **Marine Source:** A shallow source at 100 m depth.
2. **Surface Source:** A source near the seafloor at 350 m depth.
3. **Borehole Source:** A deeper source at 750 m depth.

For all scenarios, the setup includes:

- **Source:** A 25 Hz Ricker wavelet is emitted from a fixed horizontal position of 8,800 m.
- **Receivers:** A horizontal array of 100 receivers is placed at a fixed depth of 360 m, just below the seafloor, spanning most of the model's width.

For each of the three source depths, two separate elastic wave simulations are run: one using the baseline model (before injection) and another using the monitor model (with the CO_2 plume in Figure S5). The time-lapse seismic difference is then calculated by subtracting the baseline shot gather from the monitor shot gather.

The detectability of the CO_2 plume is quantified through two main analyses:

1. Qualitative Analysis (Shot gather plots): The raw time-lapse difference seismograms are plotted for each of the three source scenarios, as shown in Figures S6 and S7. These plots visually reveal the nature of the time-lapse anomaly, such as new reflections or scattered waves originating from the plume, and how its character changes with source depth.
2. Quantitative Analysis (Energy calculation): To provide a robust, quantitative measure of signal strength, the energy of the time-lapse difference is calculated. This analysis is focused on the receiver traces located directly above the CO_2 plume (between 11,300 m and 12,800 m). By calculating the mean energy and standard deviation for each of the three source depths, it is possible to determine which acquisition geometry generates the strongest and most consistent time-lapse signal, thereby indicating the optimal survey design for monitoring.

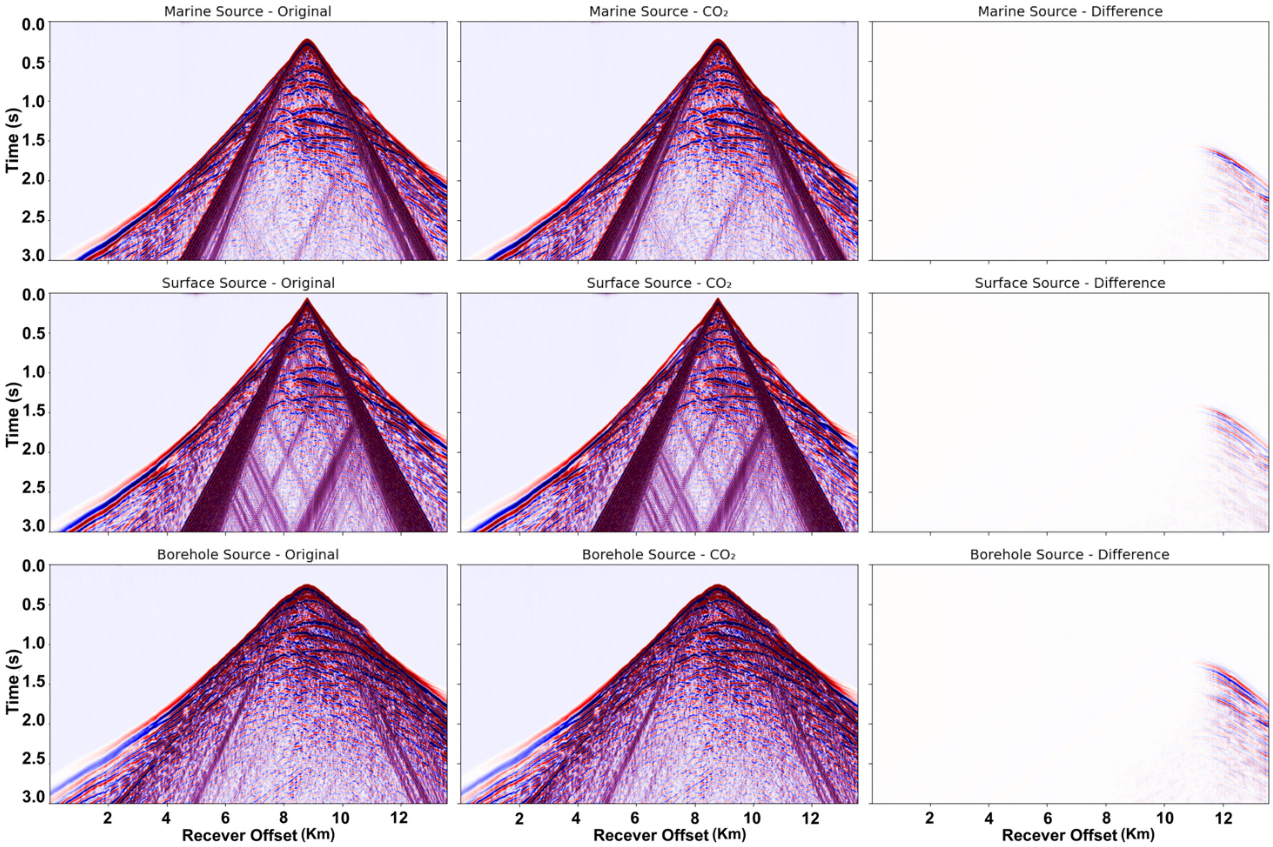

Figure S6. Comparison of simulated shot gathers for the three source configurations. Each row corresponds to a different source depth: Marine (top), Surface (middle), and Borehole (bottom). The columns display the baseline simulation without CO_2 (left), the monitor simulation with CO_2 (middle), and the time-lapse difference (right).

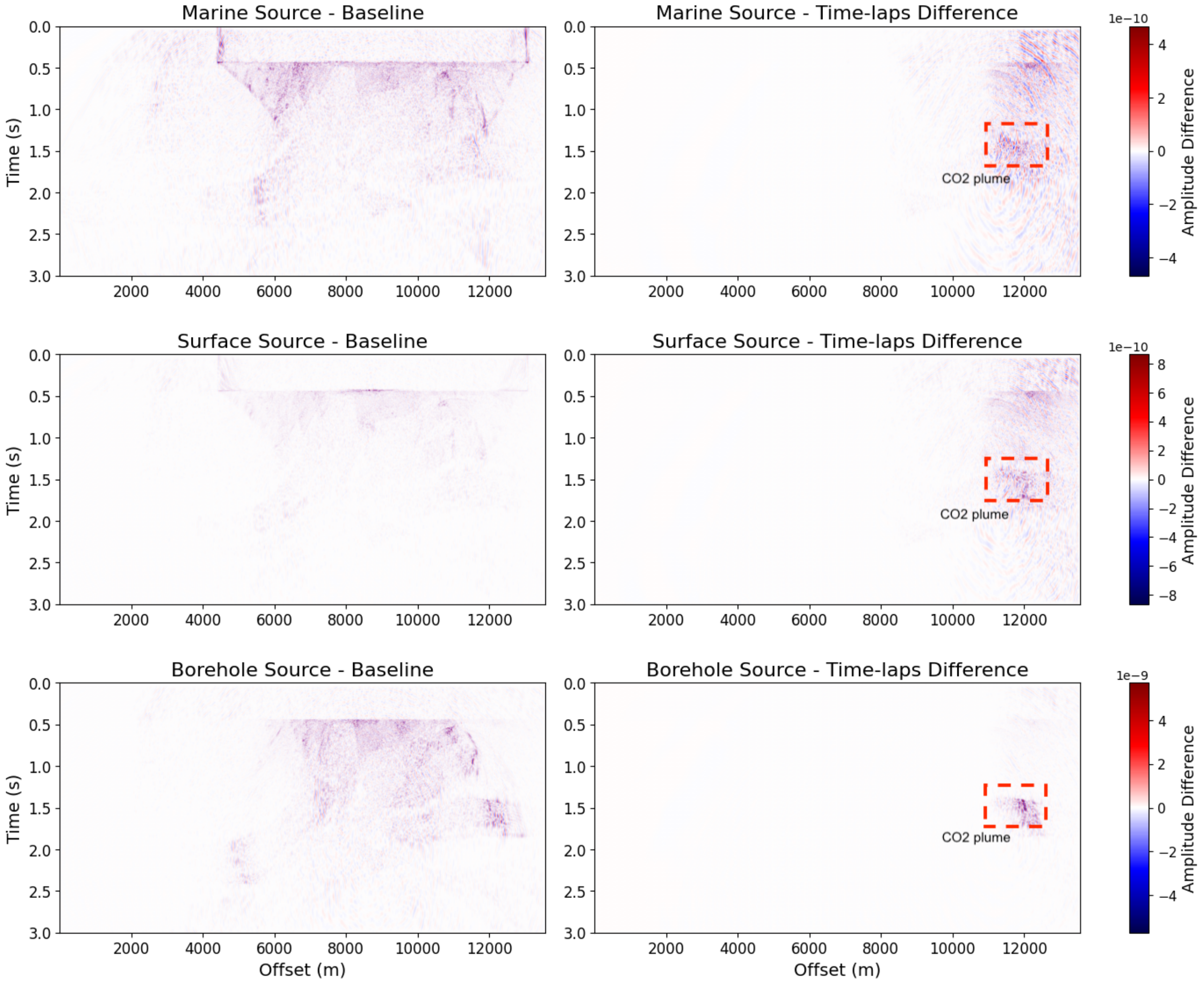

Figure S7. Simulated elastic wavefields for three source depths and their corresponding time-lapse differences. Left panels show baseline shot gathers for marine (top), surface (middle), and borehole (bottom) sources. The right panels display time-lapse differences highlighting the CO_2 plume response. The borehole source yields the strongest and most localized time-lapse signal because it bypasses the attenuating shallow layer.
